# Clinical trials of R-(-)-gossypol (AT-101) in newly diagnosed and recurrent glioblastoma: NABTT 0602 and NABTT 0702

**DOI:** 10.1371/journal.pone.0291128

**Published:** 2024-01-29

**Authors:** John B. Fiveash, Xiaobu Ye, David M. Peerboom, Tom Mikkelsen, Sajeel Chowdhary, Myrna Rosenfeld, Glenn J. Lesser, Joy Fisher, Serena Desideri, Stuart Grossman, Lance Leopold, Louis B. Nabors

**Affiliations:** 1 Department of Radiation Oncology, University of Alabama at Birmingham, Birmingham, Alabama, United States of America; 2 Departments of Neurosurgery and Oncology, Johns Hopkins University, Baltimore, Maryland, United States of America; 3 Cleveland Clinic Brain Tumor and Neuro-Oncology Center, Cleveland, Ohio, United States of America; 4 Henry Ford Hospital Hermelin Brain Center, Michigan, Indiana, United States of America; 5 Baptist Health, Boca Raton, Florida, United States of America; 6 University of Pennsylvania Department of Neurology, Philadelphia, Pennsylvania, United States of America; 7 Department of Internal Medicine, Section on Hematology and Oncology, Wake Forest University School of Medicine, Winston-Salem, North Carolina, United States of America; 8 Incyte, Wilmington, Delaware, United States of America; 9 Department of Neurology, University of Alabama at Birmingham, Birmingham, Alabama, United States of America; University of Colorado Anschutz Medical Campus School of Medicine, UNITED STATES

## Abstract

**Purpose:**

AT-101 is an oral *bcl-2* family protein inhibitor (Bcl-2, Bcl-X_L_, Mcl-1, Bcl-W) and potent inducer of proapoptotic proteins. A prior study of the parent compound, racemic gossypol, demonstrated objective and durable responses in patients with malignant glioma. AT-101 has demonstrated synergy with radiation in animal models. The objectives of trial NABTT 0602 were to determine the MTD of AT-101 concurrent with temozolomide (TMZ) and radiation therapy (RT) (Arm I) and to determine the MTD of AT-101 when given with adjuvant TMZ after completion of standard chemoradiation (Arm 2). Separately in trial NABTT 0702, the survival and response rates of single agent AT-101 were evaluated in patients with recurrent glioblastoma.

**Methods:**

In NABTT 0602 Phase I, a 3+3 design was used to define MTDs after maximal safe resection, patients with newly diagnosed glioblastoma received standard concurrent RT (60 Gy) and TMZ 75 mg/m2/day followed by adjuvant TMZ 150–200 mg/m2 days 1–5 in 28-day cycles (Stupp regimen). In Arm I, AT-101 was administered M-F during the six weeks of RT beginning 20 mg qd. In Arm 2, concurrent with each adjuvant cycle of TMZ, AT-101 was administered at a starting dose of 20 mg, days 1–21 followed by 7-day break for a maximum of 6 cycles. The PK blood samples were collected in the first three patients in each cohort of arm 1. In NABTT 0702 patients with recurrent glioblastoma received 20 mg p.o. per day for 21 of 28 days in repeated cycles to assess overall survival (OS).

**Results:**

A total of sixteen patients were enrolled on the two study arms of NABTT 0602. In Arm 1 AT-101 was escalated from 20 to 30 mg where one of six patients experienced DLT (grade 3 GI ulcer). On Arm 2 one patient treated at 20 mg experienced DLT (grade 3 ileus, nausea and diarrhea). The cohort was expanded to include seven patients without observation of DLT. PK results were consistent with drug levels from non-CNS studies. At study closure six patients are still alive. The median survival times for Arm I and Arm II are 15.2 months and 18.2 months, respectively. In NABTT 0702 fifty-six patients were enrolled and forty-three were eligible for imaging response. Sixteen patients (29%) had stable disease as best response and one partial response was observed. The median OS with single agent AT-101 was 5.7 months (95%CI: 3.8–7.6 months) for patients with rGBM.

**Conclusions:**

AT-101 can be safely administered with radiation therapy and TMZ in patients with newly diagnosed glioblastoma without toxicity unique to patients with CNS tumors. Because of toxicity observed in non-CNS AT-101 clinical trials, further dose-escalation was not attempted. The recommended dose for future studies that utilize continual AT-101 exposure is 20 mg days M-F concurrent with RT/TMZ and 20 mg days 1–21 for each 28-day cycle of TMZ. AT-101 has limited activity as a single agent in unselected patients with recurrent glioblastoma. Future trials should attempt to better understand resistance mechanisms and consider combination therapy.

## Introduction

Virtually all patients with glioblastoma die of their disease within five years. Patterns of failure studies in the temozolomide era suggest that local failure remains a component of failure in about 80% of cases [1–3]. Therefore, strategies to improve radiation response may improve overall survival. Targeting apoptotic response is one method that has been proposed to improve upon the standard temozolomide and radiation therapy (RT) regimens.

AT-101 (R-(-)-gossypol acetic acid, (Ascenta Therapeutics) is an oral bcl-2 inhibitor derived from racemic gossypol, a natural product found in cottonseed oil. Gossypol has been investigated clinically as a male contraceptive in China in over 9,000 men [4, 5]. Later in the 1990’s preclinical studies investigated the drug’s antineoplastic activity and clinical trials were performed in a variety of tumors including malignant glioma. Peter Bushunow and colleagues from University of Rochester treated 27 patients with recurrent malignant glioma with gossypol 10 mg bid until progression [6]. Two objective responses were observed including one patient with a sustained response that lasted 78 weeks.

AT-101 is the more active enantiomer of gossypol in vitro and has been investigated in clinical trials alone or in combination with chemotherapy in a variety of tumor types [7–9]. It is now recognized that AT-101 binds to the BH3 binding pocket and inhibits Bcl-2, Bcl-X_L_, Bcl-W, and Mcl-1. Other evidence suggests that AT-101 causes transcriptional upregulation of proapoptotic proteins Noxa and Puma [10]. Gossypol has also been implicated in autophagic cell death [11, 12]. Preclinical studies from a variety of tumor types suggest that AT-101 enhances both chemotherapy and radiation response [11, 13–17]. Based upon the objective responses observed with racemic gossypol in patients with recurrent glioma and preclinical data of chemoradiation enhancement in a variety of tumor types, we performed a clinical trial of AT-101 in combination with standard temozolomide and radiation therapy. The hypothesis of this approach was that AT-101 would enhance the activity of both the radiation and temozolomide leading to improved overall survival. The primary objective of this initial phase I trial (NABTT 0602) was to determine the maximum tolerated dose of AT-101 during concurrent chemoradiation (arm I) and after RT during the adjuvant phase of temozolomide (arm II). In a separate clinical trial NABTT 0702, single agent AT-101 activity was examined in patients with recurrent glioblastoma.

## Methods

### Trial schema

**Fig 1:**
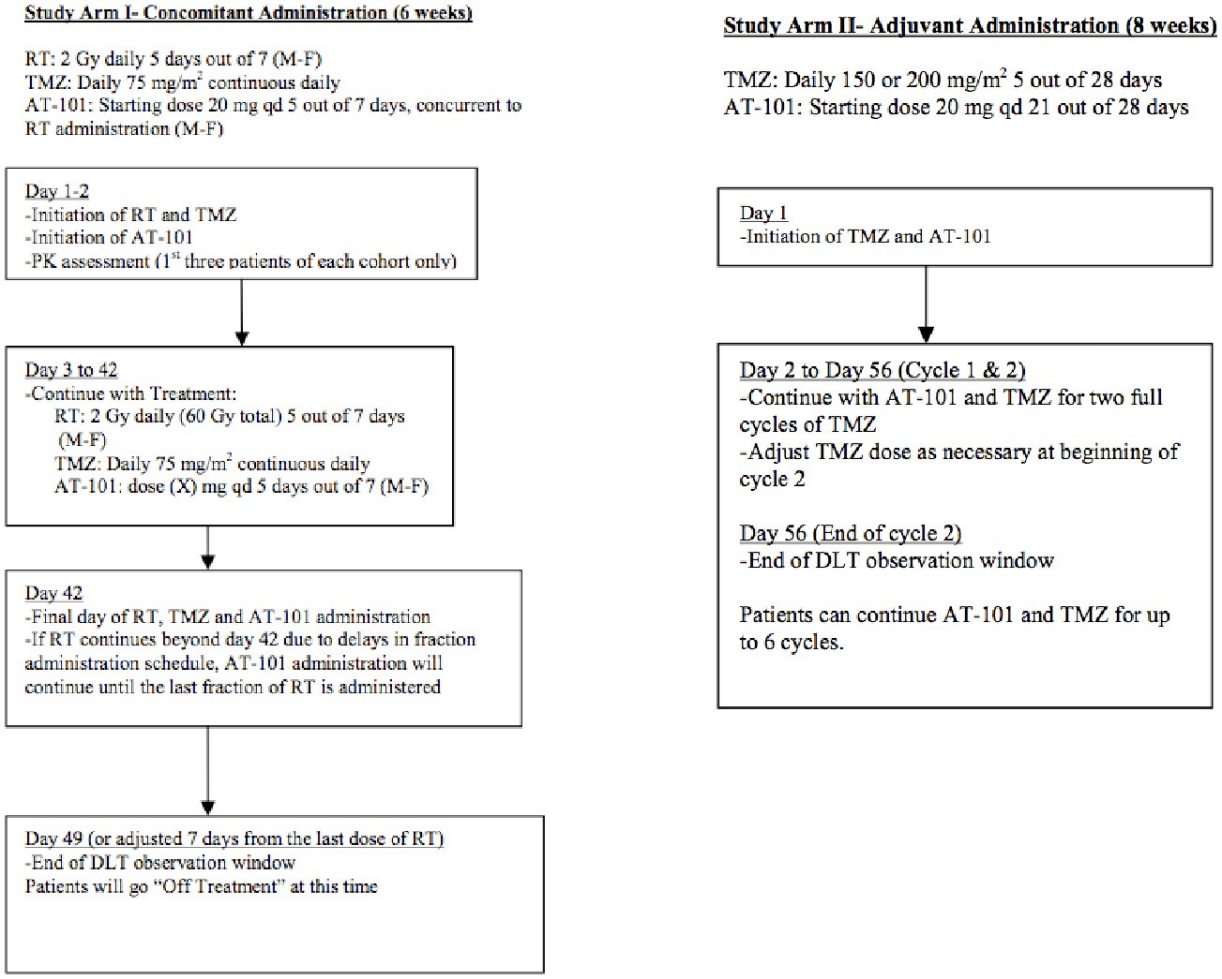
Treatment schemas for NABTT 0602 arm I and arm II. In arm I AT-101 was given five days per week during RT and concurrent with temozolomide for six weeks. In arm II enrollment and treatment with AT-101 started four weeks after completion of RT. AT-101 was administered concurrently with each adjuvant cycle of temozolomide.

In order to define the maximum tolerated dose (MTD) of AT-101 in combination with standard temozolomide and cranial RT, two phase I clinical trials (Arm I and Arm II) were designed as part of NABTT 0602. As shown in Fig 1, arm I was designed to define the MTD of AT-101 when administered Monday-Friday for six weeks during 60 Gy conformal RT and daily temozolomide. In arm II of NABTT 0602, the toxicity of AT-101 was evaluated during the adjuvant phase of temozolomide starting approximately four weeks after completion of concurrent chemoradiation.

**Fig 2:**
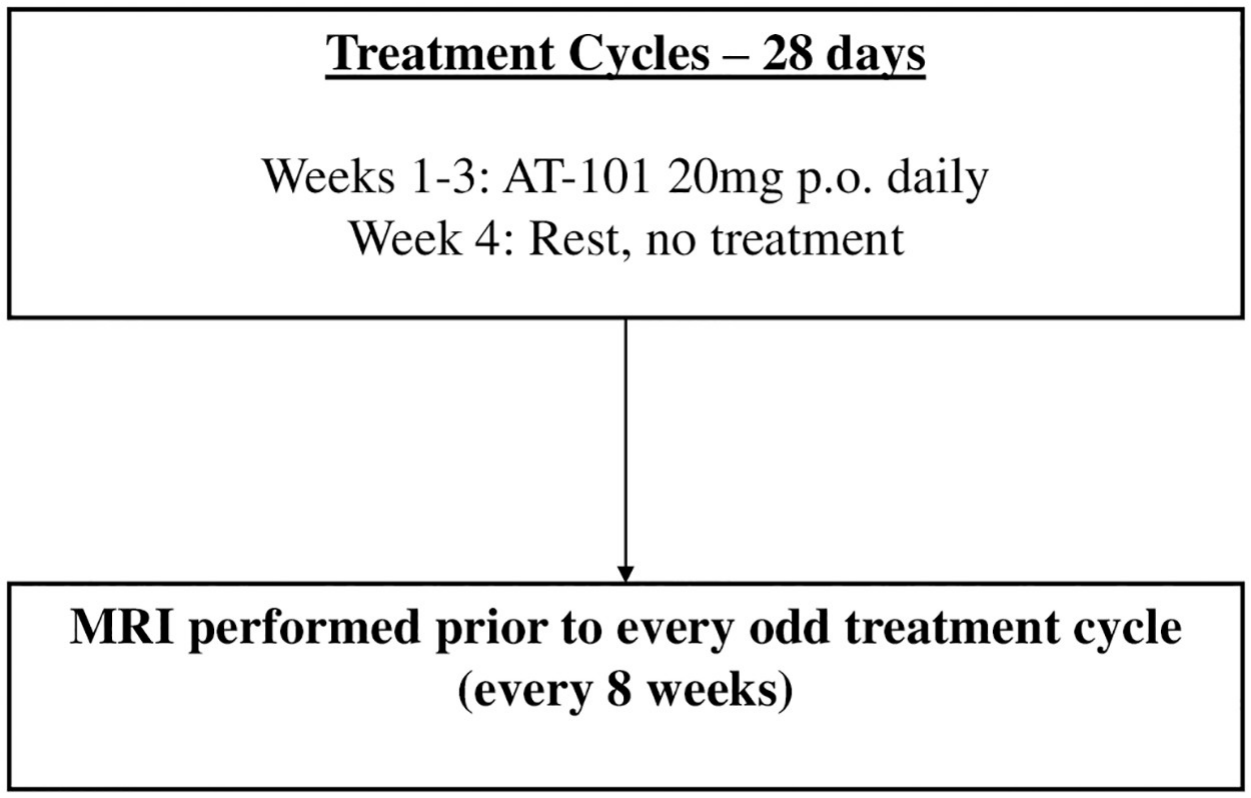
Treatment schema for NABTT 0702 testing the single agent activity of AT-101 in patients with recurrent glioblastoma. AT-101 administered 20 mg p.o. per day on days 1–21 of 28-day cycles until progression.

**Fig 3:**
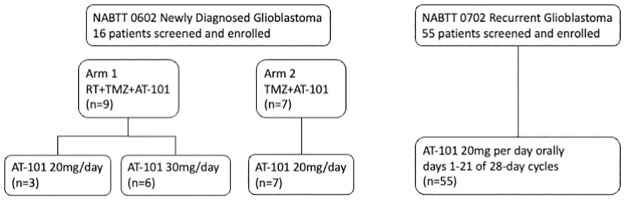
CONSORT diagram for NABTT 0602 and NABTT 0702.

NABTT 0702 was designed to test the single agent activity of AT-101 in patients with recurrent glioblastoma. The schema for this trial is shown in Fig 2. The CONSORT diagram for both trials is shown in Fig 3.

NABTT 0602 and NABTT 0702 were approved by the institutional review boards at each participating institution including the University of Alabama at Birmingham (principal investigator JF) and Johns Hopkins University, the central coordinating center for NABTT consortium.

### Eligibility

For trial NABTT 0602, eligible patients were at least 18 years of age with histologically confirmed glioblastoma. Arm I patients could not have had other prior chemotherapy, radiation therapy, immunotherapy or biologic therapy and must have completed surgery within six weeks of AT-101 administration. Arm II patients had to have completed standard temozolomide and 60 Gy conformal RT and were enrolled prior to adjuvant (post-RT) temozolomide chemotherapy. Patients had to have recovered from prior therapy with adequate hematologic, renal, and liver function including Hgb ≥ 10 g/dl, ANC ≥ 1500/mm^3^, platelets ≥ 100,000/mm^3^, Cr ≤ 1.5 mg/dl, total bilirubin ≤ 1.5 mg/dl, and AST/ALT ≤ 2.5 times institutional upper limit of normal. All patients were required to have a Mini Mental Status Exam Score of 15 or greater and Karnofsky score of 60 or greater. Patients had to be able to swallow oral medications and to comply with birth control requirements. In addition, patients had to be on a stable dose of steroids for at least five days prior to enrollment. Patients who had carmustine wafers placed at the time of surgery were not eligible for arm I, but could be enrolled in arm II. Patients taking P-450 inducing anti-epileptics or breast-feeding were not eligible. Other ineligibility criteria included pre-existing hypercalcemia (grade 2 or greater), serious gastrointestinal disorder (small bowel obstruction, malabsorptive syndromes, etc), hyperkalemia (grade 2 or greater), or grade 2 sensory neuropathy.

Trial NABTT 0702 included patients with recurrent glioblastoma. Eligibility was similar to NABTT 0602 but patients were required to be at least three months from prior radiation therapy.

### Treatment

For NABTT 0602, all patients underwent maximal safe debulking surgery. Radiation therapy was administered in two phases with three-dimensional conformal techniques utilizing the immediate postoperative MRI for treatment planning. In the initial phase, 46 Gy was prescribed to the planning target volume (PTV46) defined as the T2 or FLAIR abnormality plus a 1 cm dosimetric margin. The boost planning target volume (PTV60) was defined as the post-operative cavity plus residual T1 enhancing abnormality plus a 1 cm dosimetric margin. An additional 14 Gy was administered to the PTV60. RT was prescribed to the isocenter (ICRU reference point) with 95% isodose line enclosing the target volume or to a higher isodose volume that encompassed the PTV. The final total dose was 60 Gy administered in thirty 2 Gy fractions over six weeks. Temozolomide was administered 75 mg/m^2^/day for six weeks during RT. After a 4-week break, temozolomide 150–200 mg/m^2^ was administered days 1–5 of each 28-day cycle. On arm I the starting dose of AT-101 was 20 mg p.o. per day Monday-Friday for six weeks during RT. On arm II AT-101 was administered at a starting dose of 20 mg orally days 1–21 concurrently with each cycle of temozolomide for up to six cycles. Testing of the MGMT (O [6]-methylguanine-DNA methyltransferase) promotor was not routinely performed at the time that these trials were planned and conducted.

For NABTT 0702, single agent AT-101 was administered 20 mg per day orally days 1–21 of 28-day cycles.

### Assessments

On NABTT 0602 pharmacokinetic samples were collected as part of arm I (concomitant temozolomide arm). The first three patient of each cohort had blood samples analyzed for plasma AT-101 concentrations at six time points after first dosing over the following 24 hours. In both trials, clinical history, neurologic examination and MRI brain were performed at odd cycles (e.g. cycle 1, 3, 5, etc). Adverse event assessments and blood studies were performed with each cycle.

### Statistical analysis

Data analysis followed the intent to treat (ITT) principle. The targeted dose-limiting toxicity rate was 33% in the phase I trial to define the MTD. Three patients were treated at a dose cohort. Does escalation was in a stepwise fashion. Dose-limiting toxicity included any possibly, probably, or definitely AT-101-related Grade 3 or 4 toxicity (CTCAE 3.0). Known or reasonably suspected temozolomide hematological toxicities was not be considered dose limiting unless the treating physician considered the toxicity to be exacerbated by AT-101. With that caveat non-hematologic toxicity included grade 3 or greater nausea, vomiting, and diarrhea only if not controlled with optimal medical management. Alopecia was not considered dose-limiting. Hematologic dose-limiting toxicity included absolute neutrophil count < 0.5 x 10^9^/L and platelets <10 x 10^9^/L. Incidence of the adverse events were summarized as proportions. Baseline patient and disease characteristics were summarized descriptively. The phase II study was designed to evaluate overall survival assuming a 33% increase in median survival time (from 5 months to 6.65 months) compared to an internal historical control would be a strong efficacy signal of AT-101 to treat patients with recurrent GBM. Fifty-six patients with 52 death events were required in the phase II study to have 80% statistical power given a10% false positive rate. The survival time was defined from time of treatment start to death occurrence or censored if patient was alive at time of data analysis. Survival probability and progression-free survival were calculated using Kaplan-Meier method. All analyses were performed with the use of SAS software (version 9.4; SAS Institute).

The historical data used for the Phase II trial design and analysis of OS was obtained from NABTT internal trials which were completed in the past with the same patient population and similar inclusion and exclusion criteria to NABTT 0702. These internal historical trials were all efficacy trials for new treatments without proven to be efficacious.

NABTT 0602 and NABTT0702 are registered on ClinicalTrials.gov (NCT00540722, NCT00390403). The registration was after the studies opened for enrollment prior to the requirements of Public Law 110–85.

## Results

NABTT 0602 was started in March 2007 and enrollment was closed May 2008. Patient characteristics are shown in Table 1. The median age was 55 years. Eleven of sixteen (69%) enrolled patients had a KPS of 90–100.

**Table 1:** Patient characteristics—NABTT 0602 (Newly diagnosed glioblastoma).

	ARM I	ARM II	TOTAL
	RT+TMZ+AT-101	TMZ+AT-101	Arm I +Arm II
**Characteristics**	N = 9	N = 7	N = 16
**Age**–year			
Median (range)	55 (44–71)	55 (37–59)	55 (37–71)
**Sex**–no. (%)			
Male	6 (67)	5 (71)	11 (69)
Female	3 (33)	2 (29)	5 (31)
**Race–**no. (%)			
White	9 (100)	7 (100)	16 (100)
**Karnofsky**			
**Performance Status-** no. (%)			
90–100	7 (77)	4 (57)	11 (69)
80		2 (29)	2 (13)
70	2 (22)		2 (13)
60		1(14)	1 (6)
**Initial Procedure–**no. (%)			
Resection	6 (67)	7 (100)	13 (81)
Biopsy	3 (33)		3 (19)
**Initial Histological Diagnosis**			
no. (%)			
Glioblastoma	9 (100)	7 (100)	16 (100)
**Anticonvulsant–**no. (%)			
Yes	4 (44)	4 (57)	8 (50)
No	5 (56)	3 (43)	8 (50)

On Arm I nine patients were enrolled including three patients at 20 mg/day. In the 30 mg cohort one of six patients developed a DLT (grade 3 GI ulceration) during the toxicity evaluation phase. Arm II included seven patients. One of the first three patients developed a grade 3 ileus and four additional patients were enrolled without observation of a dose-limiting event. Overall, the most common grade 3 adverse events were gastro-intestinal (Table 2). Because of gastro-intestinal toxicity observed in other clinical trials of AT-101 utilizing continuous dosing at 30 mg or higher, further dose escalation was not attempted [16]. Similar gastro-intestinal toxicity was observed in patients receiving AT-101 monotherapy on NABTT 0702 (Table 3). Attribution to AT-101 of grade 3 and 4 events is shown in Table 4.

**Table 2:** Patient characteristics—NABTT 0702 (Recurrent patients).

CHARACTERISTICS	N = 55
**Age**–year	
Median (range)	59 (34–79)
**Sex**–no. (%)	
Male	30 (55)
Female	25 (45)
**Race–**no. (%)	
White	51 (93)
**Karnofsky**	
**Performance Status-** no. (%)	
90–100	23 (42)
80	18 (33)
70	8 (15)
60	6 (10)
**Mini-mental Score**	
Median (range)	28 (18–30)
**Prior Surgical Procedure–**no. (%)	
Resection	45 (82)
Biopsy	10 (18)
**No. of Prior Treatment**	
Median (range)	1 (1–2)
**Initial Histological Diagnosis—**no. (%)	
Glioblastoma	53 (96)
Gliosarcoma	2 (4)
**Anticonvulsant–**no. (%)	
Yes	37 (67)
No	18 (33)

**Table 3:** Grade 3 or higher toxicity combined arms I and II (NABTT 0602).

Adverse Event: N (%): Total No. of patient = 16	Grade 3
GASTROINTESTINAL: Diarrhea	1 (6)
GASTROINTESTINAL: Ileus	1 (6)
GASTROINTESTINAL: Nausea	1 (6)
Ulcer, GI—Select: Rectum	1 (6)
Absolute Neutrophil Count	1 (6)
White Blood Cell Count	1 (6)

**Table 4:** Grade 3 or 4 toxicity events with possibly, probably, or definitely related to single agent AT-101 (NABTT 0702).

Dose Level (mg)	20	Total (%)
		n = 56
Cardiac troponin T	1	2
Fatigue	2	4
GGT	1	2
Ileus, GI	1	2
Platelets	1	2

Sixty-three pharmacokinetic samples were analyzed at various time points. Peak AT-101 concentrations occurred 2–6 hours after dosing with significantly lower concentrations observed at 24 hours after dosing resulting in peak plasma concentrations of 236.7 to 744.9 ng/mL. By 24 hours the levels fell to < 30 ng/ml in over 80% of patients. There was no suggestion that temozolomide administration impacted AT-101 plasma concentrations. Full pharmacokinetic details can be found in the supplemental materials.

**Fig 4:**
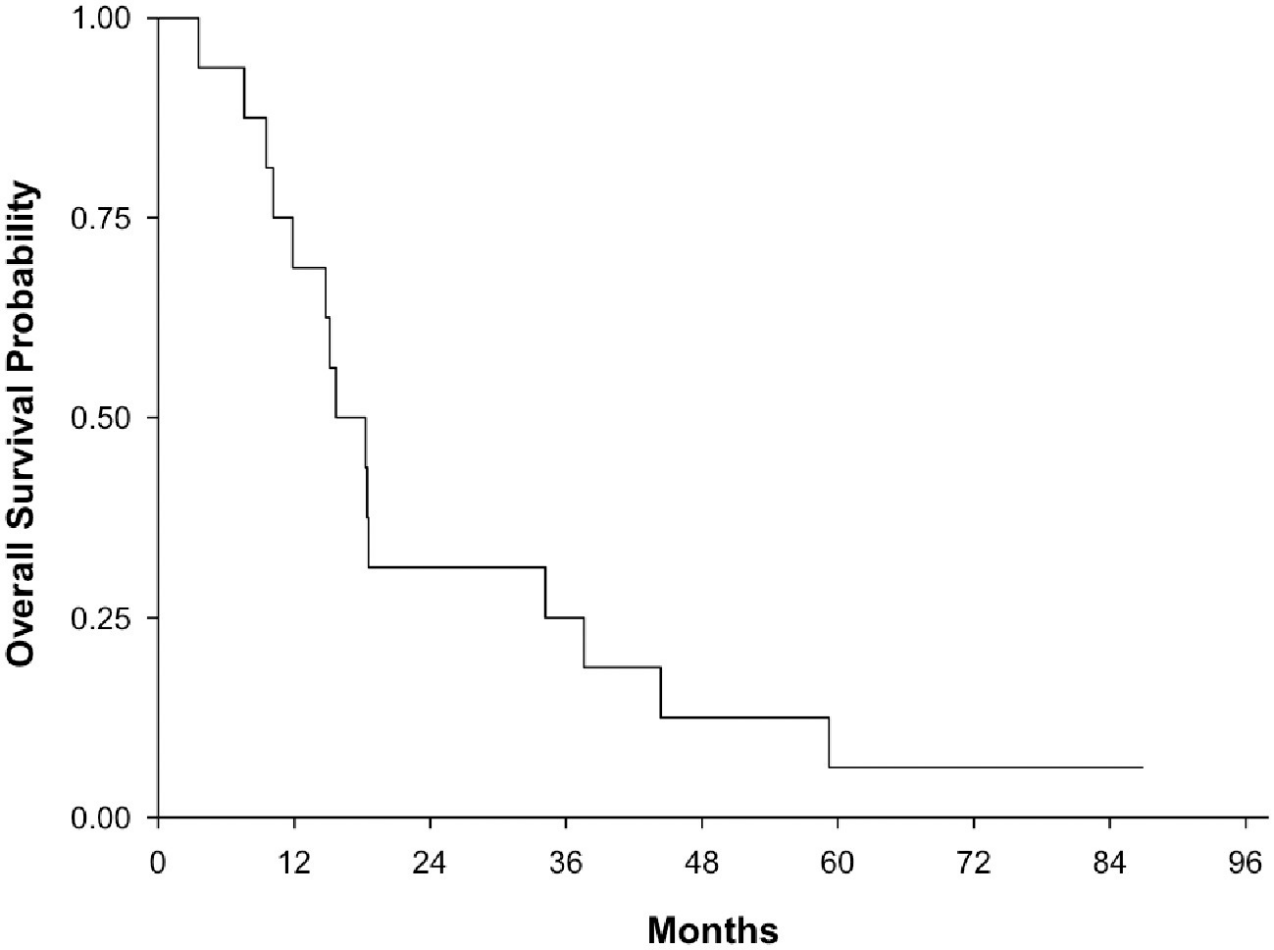
Overall survival for entire cohort including arm I and arm II (NABTT 0602).

**Fig 5:**
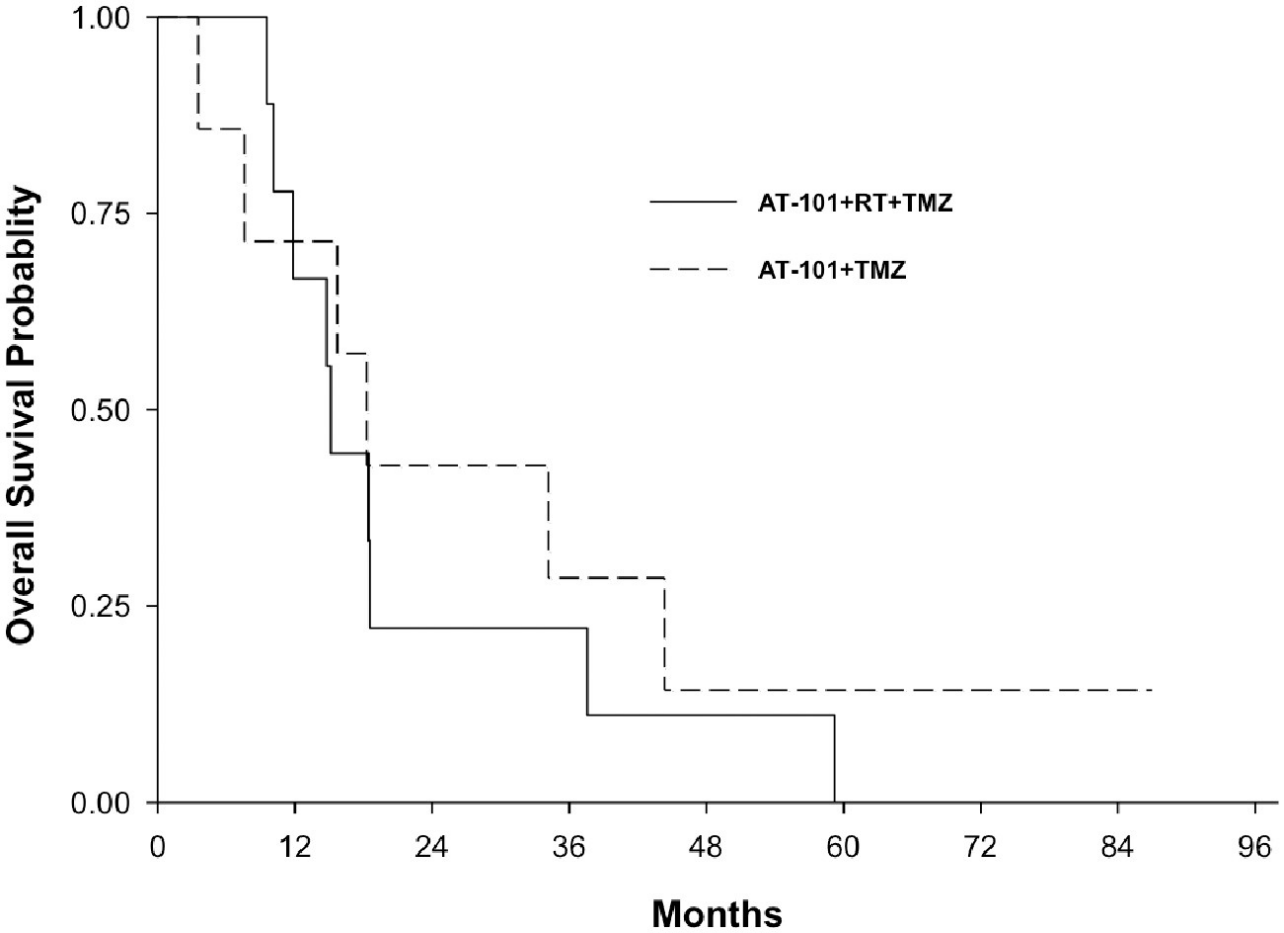
Overall survival for arm I and arm II separately (NABTT 0602).

The overall survival for both arms of NABTT 0602 is shown in Fig 4. As of April 2014, one patient was still alive. Two-year survival rate was 31.3% (95%CI: 11–59%). The median overall survival was 17 months (95% CI: 10.2–34.2 month). The median survival time for Arm I and Arm II (Fig 5) were 15.2 months (95%CI: 9.5–37.6) and 18.3 months (95%CI: 3.6–44.4), respectively.

**Fig 6:**
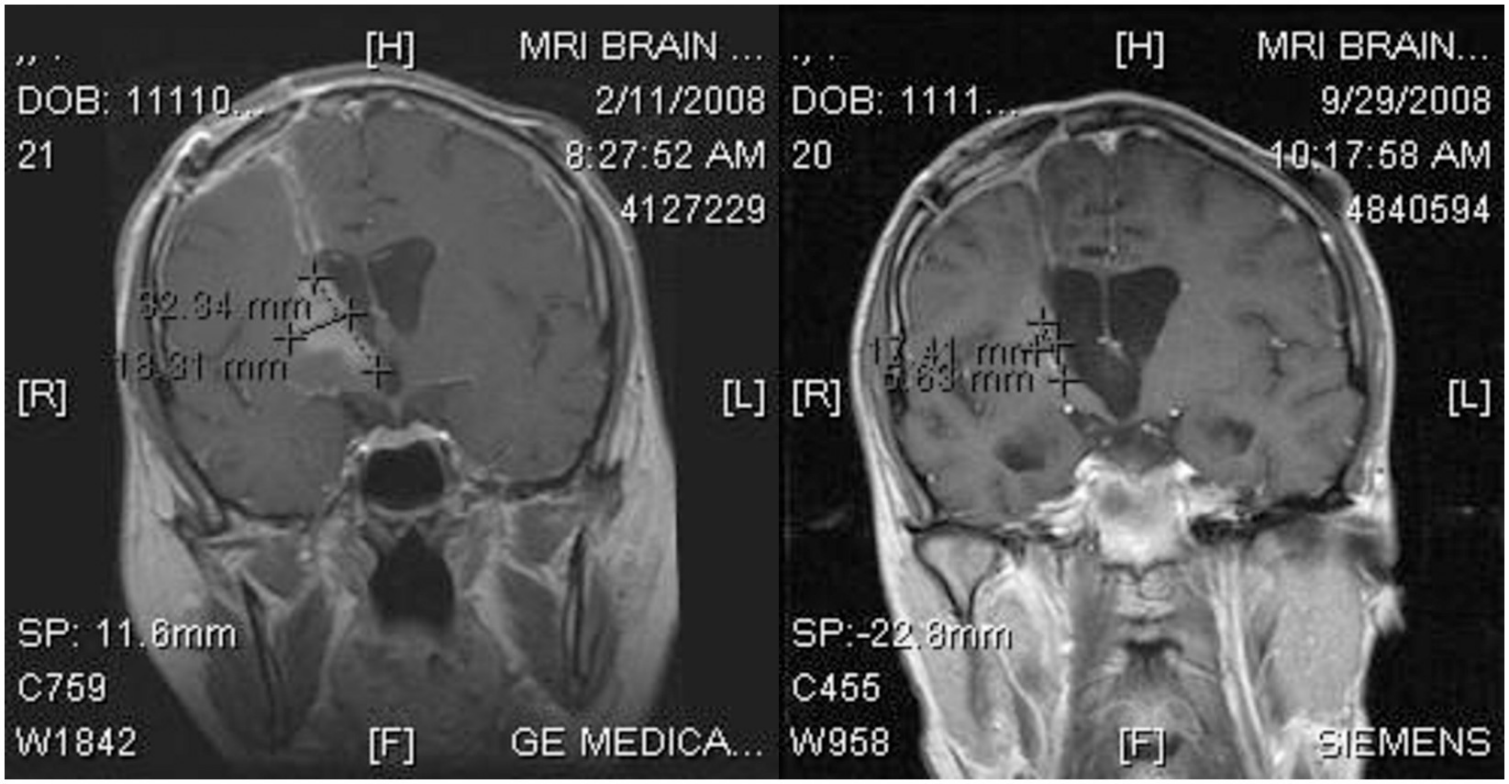
AT-101 single agent objective response in patient with recurrent glioblastoma. The MRI on the left is the post-contrast coronal image before treatment with AT-101. The MRI on the was performed with similar technique after seven months of AT-101 and demonstrates marked reduction in the enhancing abnormality.

**Fig 7:**
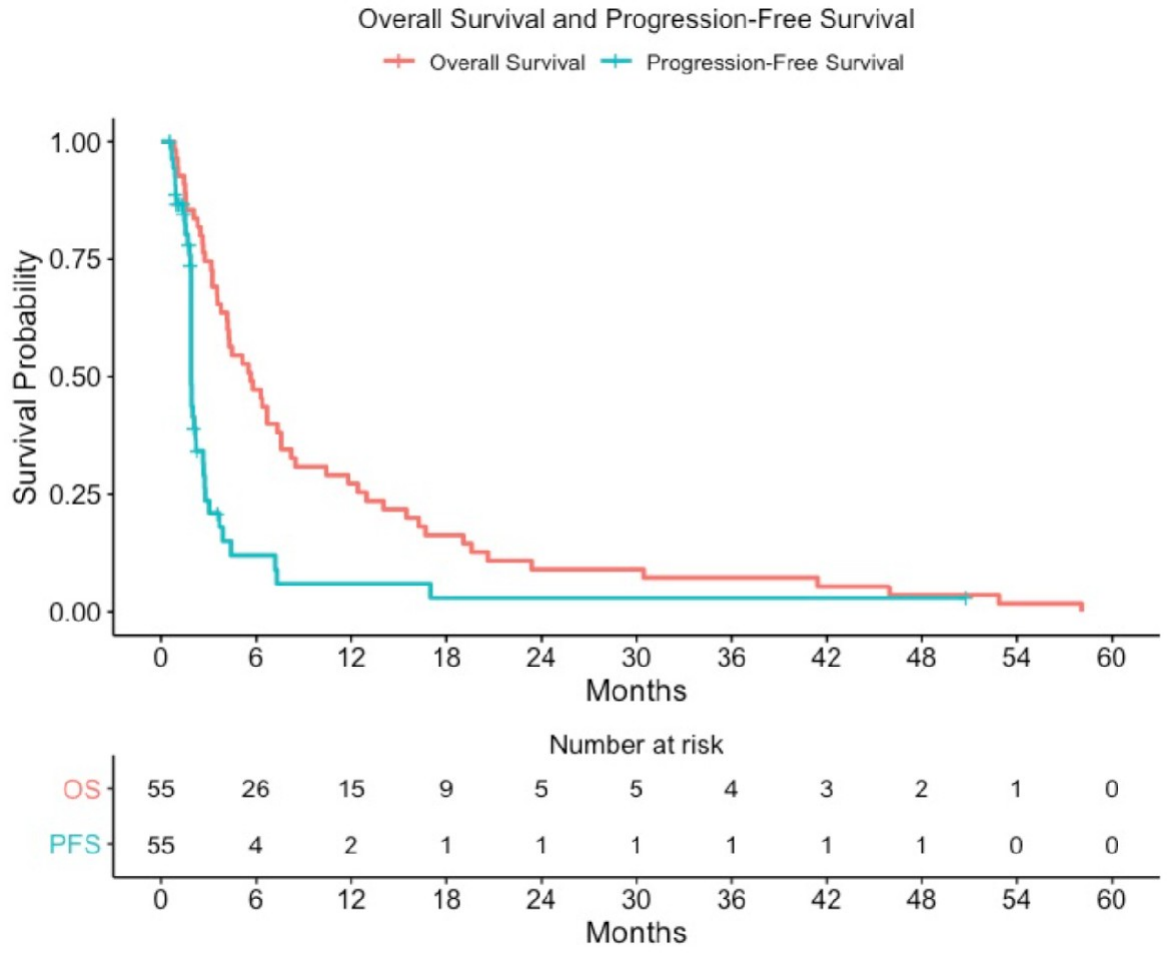
Progression-free and overall survival for AT-101 monotherapy in patients with recurrent glioblastoma (NABTT 0702).

For patients with recurrent glioblastoma enrolled in NABTT 0702, sixteen patients (29%) had stable disease as best response and one partial response was observed as shown in the MRI in Fig 6. All patients died at the time of data analysis. The progression-free and overall survival for single agent AT-101 in patients with recurrent glioblastoma is shown in Fig 7. The median survival was estimated to be 5.7 months (95%CI:3.8–7.6) and was not judged to be better than a historical control cohort. The median progression-free survival was 1.9 months (95%CI: 1.9–2.2). The 6-month progression-free survival rate was 7.3% (4/55, 95% CI: 2%-18%).

## Discussion

Pro-apoptotic anti-cancer strategies can target either the intrinsic or extrinsic apoptotic pathways. The intrinsic or mitochondrial pathway is the target of a variety of anti-cancer drugs in development including AT-101. The *bcl-2* oncogene was described in 1984. It is now recognized that Bcl-2 protects cells from death due to apoptosis. There are at least 19 human proteins that share some homology with Bcl-2 and are part of the Bcl-2 family. These proteins can be anti or pro-apoptotic and can be classified based upon the homology domains (BH1-4). Proteins with BH3-only homology are potent inducers of apoptosis. Cellular stress (e.g. radiation or chemotherapy) will enhance the induction of apoptosis. To date over twenty BH3-mimetics have been identified including agents tested in clinical trials such as AT-101 and navitoclax. Although clinical activity has been observed in patients with recurrent prostate cancer, AT-101 did not improve overall survival in a randomized trial when combined with docetaxel and prednisone for patients with hormone refractory prostate cancer [18].

In the current trials (NABTT 0602 and NABTT 0702), AT-101 was safely combined with radiation and chemotherapy for newly diagnosed glioblastoma and as a monotherapy in patients with recurrent tumors. The pharmacokinetic data suggest no unusual interactions with temozolomide that interfere with AT-101 pharmacokinetics. The M-F daily dose schedule of AT-101 during the concurrent chemoradiation was designed to provide maximal enhancement of the concurrent phase of standard chemoradiation. In future trials, dosing AT-101 2–6 hours prior to RT may provide the highest concentrations of drug to be present at the time of radiation therapy. The AT-101 toxicity observed in both arms is consistent with other studies such that no unusual CNS toxicity has been observed. Gastro-intestinal toxicity remains dose-limiting for regimens utilizing continuous dosing [16]. In other non-CNS studies, ileus and other GI toxicity was more common at 30 mg per day and higher. Based upon this information additional dose escalation was not attempted in this trial. Other BH3 mimetics (e.g. navitoclax, venetoclax) have clinical significant rates of gastrointestinal toxicity. The recommended AT-101 dose for future studies that utilize continual AT-101 exposure is 20 mg days M-F concurrent with RT/TMZ and 20 mg days 1–21 for each 28-day cycle of TMZ.

Monotherapy for patients with recurrent tumors has produced anecdotal objective responses. The prior clinical trials of the mixed enantiomer gossypol and the current monotherapy study of AT-101 provide evidence that the drug can cross the blood brain barrier and impact tumor growth as demonstrated by the objective responses observed. An ideal development pathway for new agents in glioma includes assessment of tumor concentrations and pharmacodynamics in both contrast enhancing and non-enhancing tumor regions. Until better biomarkers of efficacy are identified, clinical development would be better directed to combination therapies with radiation and chemotherapy.

Although OS results are presented, the study is underpowered to draw any conclusions regarding the efficacy of adding AT-101 to chemoradiation. Future studies would need to stratify for known prognostic factors such as MGMT promoter methylation.

## Conclusion

AT-101 can be safely administered in combination with chemoradiation for patients with glioblastoma without CNS specific toxicity. NABTT 0602 defined the recommended dose for future study of AT-101 in combination with chemoradiation for patients with newly diagnosed glioblastoma. As tested in NABTT 0702, AT-101 monotherapy activity is low and future clinical development should focus on combinatorial strategies.

## Supporting information

S1 File Clinical research protocol. (PDF)

S1 Checklist TREND checklist. (PDF)
