# Supplementary material for: Clinical trials of R-(-)-gossypol (AT-101) in newly diagnosed and recurrent glioblastoma: NABTT 0602 and NABTT 0702
Source: PLoS One. 2024 Jan 29;19(1):e0291128. doi: 10.1371/journal.pone.0291128 (PMC10824421; doi:10.1371/journal.pone.0291128)
Supplement: S1 File — (PDF) [file pone.0291128.s001.pdf]

NABTT #: 0702
NCI #: NABTT 0702

Version Date – 12/07/10
Previous Version Date – 04/20/09, 08/14/07

A Phase 2 Study of R-(-)-gossypol (Ascenta's AT-101) in Recurrent Glioblastoma Multiforme

A Protocol of the Adult Brain Tumor Consortium (ABTC)

Coordinating Center:
Sidney Kimmel Comprehensive Cancer Center at Johns Hopkins

Study Chairperson:
John Fiveash, MD
University of Alabama at Birmingham
Tel: 205-934-1432
Fax: 205-975-7546

Nursing Contact:
Thirumaine Pillay, RN
University of Alabama at Birmingham
Phone: 205-934-1842
Fax: 205-975-7546
tpillay@uabmc.edu

Pharmacy Contact:
Gopal Patil, R.Ph., Ph.D.
Sidney Kimmel Comprehensive Cancer Center
at Johns Hopkins
Phone: 410-955-8998

Pharmacology Laboratories
Jeff Supko, Ph. D.
Massachusetts General Hospital
Phone: (617) 724-1970
Email: supko@partners.org

Biostatisticians
Kathleen Lamborn, Ph. D.
University of California, San Francisco
Phone: (415) 353-2863
Email: lambornk@neurosurg.ucsf.edu

Xiaobu Ye, M.D., M.S
Johns Hopkins University
Phone: 410-614-6261
Email: xye3@jhmi.edu

IND #: TBD
Sponsor: CTEP, DCTDC, NCI
NSC #: 726190
NCI Supplied Agent: AT-101

A Phase 2 Study of R-(-)-gossypol (Ascenta's AT-101) in Recurrent Glioblastoma Multiforme

PARTICIPATING INVESTIGATORS

Cleveland Clinic Foundation
Gene Barnett, M.D.
9500 Euclid Avenue/S80
Cleveland, OH 44195
Phone: 216-444-5381

Duke University Medical Center
David Reardon, M.D.
Room 047 Baker House
Trent Drive, South Hospital
Duke University Medical Center
Durham, NC 27710
Phone: 919-668-1409

Emory University
Jeff Olson, M.D.
1365 Clifton Road, NE, Ste. B6400
Atlanta, GA 30322
Phone: 404-778-5770

Harvard Neuro-Oncology
Tracy Batchelor, M.D.
Pappas Cancer for Neuro-Oncology
55 Fruit Street, Yawkey 9E
Boston, MA 02114
Phone: 617-724-8770

Henry Ford Health System
Tom Mikkelsen, M.D.
2799 W. Grand Blvd., K-11
Detroit, MI 48202
Phone: 313-916-8641

Johns Hopkins University
Stuart Grossman, M.D.
Cancer Research Building 2, 1M16
1550 Orleans Street
Baltimore, MD 21231
Phone: 410-955-8837

M.D. Anderson Cancer Center
W.K. Alfred Yung, M.D.
Department of Neuro-Oncology, Unit 431
UT, M.D. Anderson Cancer Center
1515 Holcombe Boulevard
Houston, TX 77030
Phone: 713-794-1285

Memorial Sloan-Kettering Cancer Center
Lauren Abrey, M.D.
1275 York Avenue
New York, NY 10021
Phone: 917-432-5122

Moffitt Cancer Center
Steven Brem, M.D.
12902 Magnolia Drive
Tampa, FL 33612
Phone: 813-979-3063

University of Alabama at Birmingham
Louis B. Nabors, M.D.
Faculty Office Tower 1020
1530 Third Avenue South
Birmingham, AL 35294-3410
Phone: 205-934-1432

University of California, Los Angeles
Timothy Cloughesy, M.D.
University of California, Los Angeles
710 Westwood Plaza, Suite 1-230
Los Angeles, CA 90095
Phone: 310-825-5321

University of California, San Francisco
Susan Chang, M.D.
Neuro-Oncology Service, Box 0372
University of California, San Francisco
400 Parnassus Avenue, A808
San Francisco, CA 94143-0372
Phone: 415-353-2270

University of Pennsylvania
Myrna R. Rosenfeld, M.D., PhD
3 West Gates
3400 Spruce Street
Philadelphia, PA 19104
Phone: 215-746-4707

University of Pittsburgh
Frank Lieberman, M.D.
UPMC Cancer Pavilion
Fourth Floor
5150 Centre Avenue
Pittsburgh, PA 15232 - 1305
Phone: 412-692-2600

University of Wisconsin
Minesh Mehta, M.D.
University of Wisconsin Hospital
600 Highland Avenue
Room K4/B100
Madison, WI 53792
Phone: 608-263-8500

Wake Forest University
Glenn Lesser, M.D
Outpatient Comprehensive Cancer Center
Medical Center Blvd.
Winston-Salem, NC 27157-1082
Phone: 336-716-9527

NIH Neuro-Oncology Branch
Howard Fine, M.D.
Division of Clinical Sciences
National Cancer Institute
9030 Old Georgetown Road, Room 225
Bethesda, MD 20814
Phone: 301-402-6298

ABTC Manager

Joy Fisher
Johns Hopkins University
Phone: 410-955-3657
Email: jfisher@jhmi.edu

ABTC Data Coordinator

Serena Desideri, M.D.
Johns Hopkins University
Phone: 410-614-4400
Email: sdeside1@jhmi.edu

ABTC Protocol Development Coordinator

Megan Sims
Johns Hopkins University
Phone: 410-614-3086
Email: msims3@jhmi.edu

TABLE OF CONTENTS

1.0 OBJECTIVES 5

2.0 BACKGROUND AND RATIONALE 5

3.0 PATIENT ELIGIBILITY CRITERIA 14

3.1 PATIENT SAMPLE 14
3.2 ELIGIBILITY CRITERIA 14
3.3 INELIGIBILITY CRITERIA 15

4.0 TREATMENT PLAN 17

4.1 TREATMENT SCHEMA 17
4.2 TREATMENT REQUIREMENTS 18
4.3 DRUG ADMINISTRATION 18
4.4 MISCELLANEOUS 19

5.0 DOSE MODIFICATION FOR TOXICITY 20

5.1 DOSE MODIFICATION 20
5.2 MAJOR EVENTS 22
5.3 TOXICITY CRITERIA 22
5.4 DATA REPORTING 22

6.0 PHARMACEUTICAL INFORMATION 23

6.1 AT-101 23

7.0 PROCEDURES FOR PATIENT ENTRY ON STUDY 24

8.0 RESPONSE ASSESSMENT 25

8.1 CRITERIA FOR RESPONSE ASSESSMENT 25
8.2 ASSESSMENT OF RESPONSE 26
8.3 QUALITY ASSURANCE 26

9.0 MONITORING OF PATIENTS 27

9.1 TABLE OF REQUIRED OBSERVATIONS 27
9.2 ADVERSE EVENT DEFINITION AND REPORTING 28
9.3 COMPREHENSIVE ADVERSE EVENTS AND POTENTIAL RISKS LISTS (CAEPRS) 30
9.4 EXPEDITED ADVERSE EVENT REPORTING 32
9.5 INVESTIGATIONAL STUDIES/LABORATORY CORRELATES 35

10.0 OFF TREATMENT/OFF STUDY CRITERIA 36

11.0 STATISTICAL CONSIDERATIONS 37

11.1 OVERALL OBJECTIVE 37
11.2 PRIMARY OBJECTIVE 37
11.3 SECONDARY OBJECTIVES 38
11.4 INTERIM MONITORING 38
11.5 DECISION RULE 39

12.0 RECORDS TO BE KEPT 40

13.0 CTA 40

14.0 REFERENCES 41

15.0 INFORMED CONSENT 44

15.1 ETHICAL AND LEGAL CONSIDERATIONS 44

APPENDICES

Appendix I - NCI Common Terminology Criteria Worksheet

1.0 OBJECTIVES

Primary Objective

The primary objective of this trial is to estimate the overall survival rate associated with AT-101 in treating adult patients with recurrent glioblastoma multiforme.

Secondary Objectives

1) To assess and estimate the acute and late toxicities.

2) Tumor response rate.

3) To estimate 6-month progression free survival.

4) To explore associations of the clinical outcome (overall survival) among the changes of potential serum biomarkers, baseline tumor protein expression and gene methylation status.

2.0 BACKGROUND AND RATIONALE

Study Disease

Glioblastoma Multiforme (GBMF) is the most common primary brain tumor in adults. In the United States, approximately 18,500 new cases of primary brain tumors are expected (Jemal, 2005). Of these, approximately 52% are expected to be GBMFs (Accelerate Brain Cancer Cure website, 2005), implying the occurrence of approximately 9,500 new cases of GBMF in the U.S. in 2005. GBMF is the highest-grade primary glioma, and carries the worst prognosis. GBMF tumors are densely cellular lesions with microvascular proliferation, focal necrosis, and a high degree of cellular proliferation. A number of cellular and molecular derangements have been described in GBMF, including expression of angiogenic factors and their receptors, increased expression of a variety of oncogenes (such as the epidermal growth factor receptor, EGFR) associated with increased proliferation, and abnormalities in targets, such as p53, that are implicated in apoptosis (Louis, 2005). Clinical prognostic factors have been further assessed by a recursive partitioning analysis (Lamborn, 2004) that classifies GBMF by patient age, performance status, extent of surgical resection (subtotal vs total vs biopsy), and, for younger patients, anatomic site of the tumor (frontal lobe vs other site). This model identified patients under age 40 with frontal lobe tumors as having the best prognosis. The worst prognosis (shortest survival) was observed in this retrospective analysis for patients over age 65, or those between age 40 and 65 with poor performance status or who underwent biopsy only. For even the best prognostic group, median survivorship was only approximately 2 ½ years.

Standard initial therapy for GBMF has consisted of surgical resection to the extent safe and feasible, followed by radiotherapy (RT). Gliadel®, a carmustine-impregnated wafer that is implanted in the surgical bed, is approved in the U.S. as an adjunct to surgery and radiation in patients with newly-diagnosed high grade glioma, and as an adjunct to surgery in patients with recurrent GBMF. However,

until recently, the role of systemic chemotherapy during or after RT for newly-diagnosed patients with GBMF had not been established in a prospective, randomized trial.

Superior survival was demonstrated when temozolomide (TMZ) was added to RT after initial surgery for GBMF in a trial conducted by the European Organization for Research into the Treatment of Cancer (EORTC) and the National Cancer Institute of Canada (NCIC). In that trial (Stupp, 2005), 573 newly-diagnosed patients were randomized within 6 weeks of initial surgery to receive either RT alone or RT combined with TMZ, followed by continued TMZ for up to 6 additional months in the latter group. Median survivorship was 14.6 months for the group receiving concomitant and adjuvant TMZ vs 12.1 months for the group receiving RT alone. The observed one- and two-year survivorship were 61.1% and 26.5%, respectively, for the RT+TMZ group vs 50.6% and 10.4% for the RT-alone group. The median progression-free survival was 6.9 months for RT+TMZ vs 5.0 months for RT alone. TMZ as used in this trial was well tolerated by the patients who were assigned to receive it. Based on these results, TMZ is now approved in the U.S. for the treatment of adult patients with newly diagnosed glioblastoma multiforme concomitantly with RT and then as maintenance treatment.

In a companion study, a subset of the patients in the EORTC-NCIC study underwent an assessment of the effects of epigenetic silencing of the O^6-methylguanine-DNA methyltransferase (MGMT) gene on survival (Hegi, 2005). Methylation of the MGMT gene compromises DNA repair after administration of alkylating agents like TMZ. Consequently, the efficacy of TMZ would be expected to be enhanced in patients with MGMT gene methylation. Results from the 206 patients from the EORTC-NCIC study who were assessable for MGMT gene methylation were consistent with this expectation. The survival advantage conferred on the entire study population by TMZ was greater, and statistically significant, in the subset with MGMT gene methylation. In addition, survival in this subgroup was improved over the subgroup without MGMT gene methylation, irrespective of treatment assignment. However, for the subgroup without MGMT gene methylation, there still was a trend toward superior survivorship for TMZ+RT over RT alone, although the difference was not statistically significant.

<u>AT-101</u>

AT-101 [R-(-)-gossypol acetic acid] is the levorotatory enantiomer of gossypol, a natural substance found in cottonseeds. This orally administered BH3 mimetic inhibits the heterodimerization of Bcl-2, Bcl-xL, Bcl-w, and Mcl-1 with pro-apoptotic Bcl-2 proteins, thus lowering the threshold for apoptosis. As the racemic mixture, gossypol has demonstrated limited single-agent antitumor activity in early cancer trials and has also been administered to over 9,000 healthy volunteer men who received gossypol as a male contraceptive.

Background and Mechanism of Action

Defective regulation of apoptosis is important to cancer pathogenesis and progression and has been associated with resistance to standard therapy. Many members of the Bcl-2 family of apoptosis-related genes are differentially expressed in various malignancies, and overexpression of the protein products of some Bcl-2 genes has been reported to correlate with adverse prognosis in certain malignancies. The Bcl-2 family of proteins includes proapoptotic members (*e.g.,* Bax, Bak, Bad, Bid, Bim) as well as those that act to suppress apoptosis (*e.g.*, Bcl-2, Bcl-xL, Bcl-W, Mcl-1). The ratios and interactions of these anti- and pro-apoptotic proteins determine the sensitivity or resistance of cells to various apoptotic stimuli, and alterations in the amounts of these proteins have been associated with a variety of pathologic conditions characterized by either too much or too little cell death (Letai, 2003). The pro-

and anti-apoptotic proteins may titrate each other with the relative concentrations of the opposing sub-family members determining cell death or cell survival (Heiser *et al*., 2004). In both types of proteins, there are certain regions that are homologous (Bcl-2 homology or BH regions) which control the ability of the members of the Bcl-2 family to bind to each other to form homo- and heterodimers. Many data indicate that Bcl-2 (and Bcl-xL) bind to the BH3 domains of pro-apoptotic family members, sequestering them and thereby inhibiting their ability to promote cell death. Peptide and non-peptide BH3 mimetics have been shown in laboratory studies to inhibit the heterodimerization of Bcl-2 or Bcl-xL to pro-apoptotic BH3 family members with the attendant expected effects of increasing mitochondrial permeability and cytochrome c release, activation of effector caspases, and apoptosis. AT-101 is a BH3 mimetic that inhibits the heterodimerization of Bcl-2, Bcl-xL, Bcl-W, and Mcl-1 with pro-apoptotic members of the Bcl-2 family at submicromolar affinity (Investigator's Brochure, 2006).

Nonclinical Studies

In vitro – Efficacy. Results of nonclinical *in vitro* and *in vivo* pharmacology studies with racemic gossypol and R-(-)-gossypol determined that the primary antitumor potential is found in the levorotatory enantiomer (Shelley *et al.* 1999), now under development as AT-101. Nonclinical studies have focused on characterizing the mechanism of action of the agent and comparing the *in vitro* characteristics of the two enantiomers, R-(-)- and S-(+)-gossypol, as well as evaluating the growth-inhibitory ability of (-)-gossypol or AT-101 in various tumor types. The racemates have shown similar binding affinities for Bcl-2 and Bcl-xL proteins but differing antiproliferative potencies in the National Cancer Institute (NCI) 60-cell screen (Investigator's Brochure, 2006). The average 50% growth inhibition (GI_{50}) over the entire NCI panel of cells was 0.57 mcM for R-(-)-gossypol, 2.08 mcM for the racemic mixture, and 14.45 mcM for S-(+)-gossypol, indicating that the R-(-) enantiomer is primarily responsible for the anticancer properties of gossypol.

In both breast and head and neck cancer cell lines that overexpress Bcl-2/Bcl-xL proteins, R-(-)-gossypol induced apoptosis in a dose-dependent manner, but there was little effect in cell lines with low levels of these proteins. S-(+)-gossypol was comparatively less effective in producing apoptosis. *In vitro* studies have also shown that (-)-gossypol is able to induce cell death in a pediatric tumor, rhabdomyosarcoma (Fuh *et al*., 2006). AT-101 has shown the ability to induce apoptosis in a time- and dose-dependent manner in chronic lymphocytic leukemia (CLL) cells (Prada *et al*., 2005) and has also shown significant *in vitro* activity in myeloma, in cell lines and primary patient cells as well as in cell lines resistant to conventional agents used to treat myeloma (Kumar *et al*., 2005).

Nonclinical Combination Studies

In vitro studies of combination treatments have shown that R-(-)-gossypol synergizes with docetaxel in suppressing the growth of breast cancer cells (*e.g.*, MDA-231) (Investigator's Brochure, 2006). R-(-)-gossypol has also produced a synergistic response to irradiation in prostate cancer cells (PC-3). The diffuse large cell lymphoma cell line WSU-DLCL$_2$ as well as fresh cells from a lymphoma patient exhibited significant growth inhibition when exposed to (-)-gossypol (Mohammad *et al*., 2005). In these studies, a substantially increased cell kill resulted when the cells treated with (-)-gossypol were then exposed to the CHOP (cyclophosphamide-Adriamycin-vincristine-prednisolone) regimen. AT-101 combined with rituximab has shown synergistic cytotoxicity *in vitro*, with the effect apparent in various CLL cell types (James *et al*., 2005).

A nude mouse model of human breast cancer (MDA-MB-231) was used to evaluate the antitumor effect of oral R-(-)-gossypol (7.5, 15, or 30 mg/kg daily for 4 weeks) with or without combination treatment with docetaxel [7.5 mg/kg given as a weekly intravenous (IV) injection for 3 weeks] starting on day 2 of R-(-)-gossypol treatment (Investigator's Brochure, 2006). Overall, there was approximately 90% tumor growth inhibition in the combination groups compared to the control group. A subset of animals given a second cycle of combination treatment had an average 50% tumor volume reduction, while tumors continued to grow in the control animals (docetaxel only). In a similar study in a prostate cancer model (PC-3), each agent alone produced 70% tumor inhibition, but the combination treatment led to over 90% tumor growth inhibition. Of note, 9 of 14 PC-3 tumors regressed completely following combination treatment with only scar tissue remaining. An additional study in the PC-3 prostate cancer model using cisplatin instead of docetaxel produced less dramatic results overall, but the synergy of the combination was even more striking.

Irradiation combined with R-(-)-gossypol in a murine PC-3 xenograft model at a point when tumors were 100 mm^3 in volume led to significantly greater tumor growth inhibition (96.6%) than with either irradiation (63.1%) or R-(-)-gossypol (4.2%) alone (Xu *et al.* 2005). When starting tumor volumes were larger, similar results were seen although complete tumor regression did not usually result. These authors reported that histologic analysis verified significantly enhanced apoptosis in tumors treated with combination treatment, while immunohistologic staining showed substantially decreased tumor angiogenesis as well.

Several schedules of AT-101 administration were studied in a murine model of aggressive B-cell lymphoma (RL-DLBCL) with the result that the greatest tumor volume decrease and least toxicity was observed when 35 mg/kg was administered daily by the subcutaneous (SC) route during 2 out of 4 weeks (Paoluzzi, *et al.,* 2005). While tumor volume reduction occurred with single agent AT-101, there was marked improvement when cyclophosphamide and rituximab were added to the regimen.

Activity of AT-101 in small cell lung cancer (SCLC), alone and in combination with standard chemotherapy agents, has been demonstrated using *in vitro* and *in vivo* models. Using a panel of SCLC cell lines (H417, DMS 79, H82 and H-69), dose ranging experiments were conducted with AT-101 as single agent or in combination with cytotoxic drugs (topotecan, etoposide, carboplatin). Anti-tumor activity was demonstrated with AT-101 as a single agent, with an IC_{50} in H82, DMS, H 417, and H69 cells of 1.94 mcM, 3.13 mcM, 3.98 mcM, and 6.84 mcM, respectively. AT-101 also demonstrated a strong synergy with cytotoxic drugs (combination index >0.3) using the NCI-H446 cell line. The combination index with topotecan, etoposide, and carboplatin was 0.4, 0.6, and 0.8, respectively.

The *in vivo* anti-tumor activity of AT-101 alone and in combination with topotecan was tested in immunosuppressed mice using H69 cells in a xenograft model. Mice with established tumors were treated with vehicle, AT-101 at 30 mg/kg/day × 28 days, topotecan 2 mg/kg/day × 5 days every 14 days, or the combination. AT-101 significantly suppressed tumor growth alone and demonstrated synergy in combination with topotecan in this model.

Nonclinical Pharmacology

Pharmacologic studies in animals using orally administered ^{14}C-radiolabeled racemic gossypol showed that radioactivity was slowly absorbed from the gastrointestinal tract, and that peak tissue radioactivity

levels varied among species, ranging from 1 day to 9 days post-dosing. The $t_{½}$ for ^{14}C in the blood was 31 hours in mice, 16.5 hours in rats, 45 hours in the dog, and 11 hours in the monkey, values that appear to correlate with the toxicity profile of gossypol in these species (Tang *et al.*, 1980). Radioactivity was consistently found in the feces in all species tested, with the high concentrations of gossypol seen in bile suggesting biliary circulation of the agent between the liver and the intestine. Although moderate levels of gossypol were detected in the kidney during tissue distribution studies, the amount of gossypol-associated radioactivity excreted in urine was consistently limited to 2 to 3% of the administered dose suggesting nearly complete reabsorption of the agent in the kidney.

Nonclinical Toxicology

Repeat dose toxicity studies of R-(-)-gossypol have been conducted in cynomolgus monkeys given 1.5, 4, or 5 mg/kg/day for 4 weeks (Heywood, 1988). Dose-related gastrointestinal signs were seen at all dose levels, and body weight was affected at the two higher doses. The only consistent pathologic finding was reduced liver weights. Repeat dose studies in rodents resulted in decreased body weight/weight gain, decreased food consumption, slightly increased ALT, low cholesterol, and increased inorganic phosphorus and chloride. The expected morphologic changes in the male reproductive organs included focal tubular atrophy and/or testicular atrophy.

In dogs, gossypol appears to accumulate in the cardiac tissues; in addition, this species has a long terminal half-life and prolonged excretion of gossypol. Daily doses of 2 mg/kg for 63 days, 4 mg/kg for 71 days, and 5 mg/kg for 40 days were associated with mortality (Sang *et al.*, 1980). These dogs showed dose-related electrocardiogram (ECG) abnormalities as early as 4 weeks after beginning treatment. The deaths were due to cardiac toxicity and were accompanied by marked changes to ECGs; the ECGs of dogs that survived gradually recovered to normal, suggesting that cardiac toxicity in dogs may be reversible. Other toxicities seen in dogs included decreased food consumption, distended abdomen, general atrophy, and listless behavior.

Clinical Experience

Racemic Gossypol

Racemic gossypol has been evaluated as an antitumor agent with published results available on over 100 patients with advanced cancer (Stein *et al.*, 1992), adrenal cancer (Flack *et al.*, 1993), malignant glioma (Bushunow *et al.,* 1999), or metastatic breast cancer (Seidman *et al.,* 1999). Doses in these studies ranged from 30 to 180 mg weekly or from 20 to 70 mg daily. Reported side effects in these trials were mild to moderate and consisted of gastrointestinal effects (nausea, vomiting, diarrhea, transient ileus, anorexia) as well as transaminitis, fatigue, peripheral edema, and rash. Hypokalemia and hematologic adverse events (AEs) were rare in these cancer patients. Partial responses (PRs) were seen in adrenal cancer and glioma with minor responses and/or stable disease reported in all studies.

As a component of cottonseed meal and oil, racemic gossypol has been consumed as part of the human diet or as an ingredient in folk medicines for many years (Investigator's Brochure, 2006). Its use as an antifertility agent was suggested by the observation that when Chinese villagers substituted the less expensive cottonseed oil for the more expensive soy oil, a period of extensive infertility resulted. Human experience in clinical studies with racemic gossypol is extensive, including over 9000 Chinese men as well as smaller numbers of men of different ethnic origins who received the agent in contraceptive studies. Since the 1970s, racemic gossypol has also been used as an oral treatment for

endometriosis, functional bleeding of the uterus, and uterine myoma, and is currently approved by the Chinese State Food and Drug Administration for these indications. In contraceptive studies with gossypol, a dose of 20 mg daily for 75 days was effective but resulted in irreversible infertility in 10-15% of the men. Mild hypokalemia was also reported, but in less than 0.75% of patients on a large Chinese trial, primarily in those subjects from regions with low pretreatment serum potassium levels; this AE has rarely been reported in studies conducted outside of China. Other AEs associated with gossypol treatment included fatigue, gastrointestinal symptoms, decreased libido, and prolonged infertility.

<u>AT-101</u>

A preliminary report on the initial clinical trial of AT-101 included information on 25 patients with advanced solid tumors or lymphoid malignancies. These patients received oral doses of the agent ranging from 5 to 40 mg daily or 30 mg twice daily (BID) given during 21 of every 28 days. AT-101 is being investigated in several phase 1and 2 clinical trials, both as a single agent and in combination with standard chemotherapies. As of November 2006, approximately 155 patients have been enrolled various trials (Personal Communication. Ascenta; Saleh *et al*., 2005). Most patients have received AT-101 once or twice daily, every day for 21 out of every 28 days, in these trials. Alternative schedules are also being evaluated, which include drug "holidays" and pulsed dosing.

Preliminary data show that the most common AEs on a once daily schedule have been gastrointestinal and grade 1/2 fatigue. The gastrointestinal AEs include nausea, vomiting, abdominal pain, abdominal discomfort, diarrhea, constipation, and small bowel obstruction (SBO). The most common serious AEs reported include nausea, vomiting, SBO, and ileus, especially after several months of continuous dosing. Elevated troponin has been reported in 10 patients. Four of these were in conjuction with serious AEs. Four patients had isolated elevations of troponin, and three others had elevated troponin not considered clinically significant by the investigator. There have been 13 instances of cardiac rhythm abnormalities, of which 3 were possibly related to AT-101. Two AEs were arrhythmias, one ventricular arrhythmia (possibly related), one palpitations, one premature atrial contraction (possibly related), three premature ventricular contractions (one possibly related), three sinus tachycardia, and three tachycardia.
In the initial Phase 1 trial, DLTs were reversible grade 4 elevated ALT/AST at daily doses >40 mg/day (Saleh *et al*., 2005). The principal AEs seen with daily dosing include grade 1 nausea, diarrhea, and fatigue, but one of six patients receiving 40 mg AT-101 daily experienced grade 4 hypokalemia and grade 3 nausea. Stable measurable disease and a reduction in circulating lymphocytes from about 33,000/dL to 22,000/dL were reported in one patient with CLL after 5 cycles at 10 mg AT-101 daily. Of interest, this patient's lymphocyte count consistently rose during the week off therapy, then decreased once AT-101 was restarted. Other evidence of efficacy has been provided by three patients with non-small cell lung cancer (NSCLC) who remained on study with stable disease (SD) for more than 72 to 123 days; in addition, SD (66+ days) has been reported in a patient with a parotid tumor treated at 40 mg daily AT-101.

Additional Clinical Studies with AT-101

AT-101 is being investigated in several Phase 1-2a clinical trials as a single agent or in combination with standard agents for the treatment of patients with advanced solid tumors, lymphoma, or chronic lymphocytic leukemia. As of November, 2006, approximately 155 patients have been enrolled on 8 Phase 1/2 trials. Most patients have received AT-101 once or twice daily, every day for 21 out of every 28 days, in these trials. Total daily AT-101 doses for these patients have been 5-60 mg. The majority of patients have been treated with AT-101 on a daily schedule; however, alternative schedules are currently being tested.

Clinical trials conducted with AT-101 are ongoing; however, preliminary safety data show that the most common AEs, when AT-101 is administered on a once daily schedule, have been gastrointestinal (GI) and grade 1/2 fatigue. The GI AEs include nausea, vomiting, abdominal pain, abdominal discomfort, diarrhea, constipation, and small bowel obstruction (SBO). The most common SAEs reported include nausea, vomiting, SBO, and ileus, especially after several months of continuous dosing. While some of these SAE reports have been unrelated to AT-101 administration and attributed to disease progression, the majority have been related to AT-101 and have resulted in the hospitalization of research subjects. Reversible elevation of ALT and AST were dose-limiting at doses greater than 40 mg/day, administered daily for 21/28 days, in the initial Phase 1 trial.

Following periodic safety reviews, Ascenta has modified the dosing schedule of single-agent AT-101 to include a drug 'holiday' and has reduced the dose of single-agent AT-101, when administered daily, to 20 mg/day for 21/28 days. Additionally, Ascenta is also investigating shorter periods of exposure (pulse dosing) of AT-101 as a single agent and in combination with standard agents. The results of these changes were only recently made and the effect of these new doses and schedules on gastrointestinal AEs and SAEs has not yet been fully determined.

As of November 2006, there have been 32 SAEs reported after treatment with oral AT-101. Twenty-four patients have had events that represent, or may represent, small bowel obstruction/ileus. Several of these events were considered by the investigator to be unrelated to AT-101, but nonetheless prompted brief hospitalization and interruption of AT-101 dosing, with resolution of symptoms 2-10 days later. Two patients were successfully re-challenged at a lower dose. These events tended to occur at higher doses and typically had an onset later than 4 weeks after the first dose of AT-101. The dosing regimens associated with these events involved continuous daily dosing or dosing for 21 out of 28 days. It is not clear how recent modifications in the dose and schedule will affect the incidence and severity of these or similar events. Further investigations into other doses and schedules of AT-101 are underway.

Based on IND-related discussions with the FDA prior to initiating clinical trials with AT-101, weekly troponin and ECG testing had been included in the initial Phase 1 study and subsequent Phase 1 and 2 clinical trials. This testing was included because of data reported in the literature that dogs treated with gossypol developed cardiac damage. There have been a few patients with elevations in troponin and/or myocardial vascular complications while on clinical trials with AT-101, including 3 patients with SAEs related to the cardiovascular system. As of the most recent periodic safety review with data of data cut-off of 31 July 2006, there have been at total of ten patients that have experienced elevated troponin levels on studies with AT-101. Four of the elevated troponin levels were in conjunction with serious adverse events. Two patients had elevated troponin levels at baseline and prior to AT-101 dosing. One of these patients had a negative cardiac evaluation but the treating investigator elected to remove the patient from study. The other patient had three weeks of normal troponin values during treatment with AT-101, however, was discontinued from the study per the patient's request. Four other patients had

isolated elevations of troponin to 0.3-0.8 ng/mL (upper limit of normal, 0.2 ng/mL). One of these patients had a single elevation of serum troponin after one week of AT-101 at 50 mg/day, with no associated cardiac findings, and continued to receive AT-101 for one complete cycle with no reported cardiac AEs and with normalization of the troponin level. The three other elevations were not considered clinically significant by the investigator. These patients did not have cardiac related clinical findings, and the troponin levels were found to be normal upon repeat testing within one week later.

ECGs were performed on all patients prior to enrollment and weekly during the treatment period with AT-101on the initial Phase 1 study and each month on subsequent studies. The ECGs were assessed as either normal, abnormal but not clinically significant, or abnormal but clinically significant with comments by the investigator. As of 31 July 2006, there have been no reports of QTc prolongation reported as AEs or SAEs.

There have been 13 adverse events categorized for cardiac rhythm abnormalities. Only 3 of these were reported as possibly related to AT-101. Of the 13 reports, 2 were for arrhythmias, 1 was for a ventricular arrhythmia and was possibly related, 1 was for palpitations, 1 was for PAC and was possibly related, 3 were for PVCs with 1 possibly related, 3 were for sinus tachycardia, and 3 were for tachycardia.

<u>Pharmacology</u>
A clinical study in healthy male volunteers compared the pharmacokinetics (PK) of a single 20 mg dose of R-(-)-gossypol to that of S-(+)-gossypol (Wu *et al.*, 1986). The area under the concentration-time curve (AUC) for R-(-)-gossypol was approximately one-fifth that of S-(+)-gossypol, and the terminal $t_{½}$ for R-(-)-gossypol was about 30- to 60-times shorter than S-(+)-gossypol or racemic gossypol, respectively.

In the initial AT-101 trial, PK studies using an enantiomer-specific LC/MS method showed that PK values for AT-101 are identical to those of the (-) enantiomer when administered as racemic gossypol, and there was no evidence of interconversion of the enantiomers (Investigator's Brochure, 2006). Peak plasma concentrations increased with dose, and at the recommended phase 2 dose of 40 mg per day, the mean peak plasma concentration was 1653 +/- 1561 ng/mL (3.2 +/- 3.0 mcM) at 4 hours post-dosing with an approximate plasma half-life of 4 hours. Although there were large variations between patients in some individual parameters, these results are consistent with those obtained in earlier studies.

Drug Interactions
In vitro studies of the potential for racemic gossypol acetic acid to either inhibit or induce CYP450 isoenzymes determined that at clinically relevant concentrations of the agent up to 2 mcM, CYP isoforms (1A2, 2A6, 2B6, 2C9, 2C19, 3A4) and UDGPT showed only slight effects (Investigator's Brochure, 2006). These data suggest that minimal if any drug interactions are likely to occur when racemic gossypol or its levorotatory enantiomer, AT-101, are given at clinically relevant concentrations.

<u>Rationale</u>

The primary clinical rationale for studying AT-101 in patients with glioblastoma multiforme comes from a clinical trial of racemic gossypol performed by Bushunow and collegues from University of Rochester (Bushunow 1999). In this clinical trial racemic gossypol was administer at 10 mg bid to 27 patients with gliomas that had relapsed after radiation therapy. Histologic tumor types include fifteen

patients with glioblastoma multiform, eleven patients with anaplastic astrocytomas, and one patient with a relapsed low grade glioma. Treatment was continued until progression. Two of the 27 patients developed a partial response radiographically and four additional patients had stable disease for at least eight weeks. In one patient a partial response was maintained for 78 weeks. The in vitro cytotoxicity of AT-101 suggests that it will have greater clinical activity than racemic gossypol. The New Approaches to Brain Tumor Therapy (NABTT) CNS consortium activated a clinical trial of AT-101 in newly diagnosed glioblastoma multiforme in combination with standard radiation therapy and temozolomide chemotherapy. In this clinical trial we proposed to define the efficacy and toxicity of AT-101 as a single agent in patients with relapsed glioblastoma multiforme.

Correlative Studies Background

Apoptosis can be triggered through either the intrinsic (mitochrondrial) or extrinsic (receptor) pathways. Either of these pathways can lead to caspase activation and result in apoptosis. Regulation of the intrinsic and extrinsic pathways is not completely mutually exclusive. Altered signaling of either pathway can alter the threshold for apoptosis in a variety of tumor types including gliomas (Song). For this reason we will focus correlative studies on regulators of both the intrinsic (Bcl-2, Bcl-XL, Mcl-1, Bax, Bak) and extrinsic (FLIP, survivin, XIAP) pathways. Analysis of Bcl family members in serum has been previously performed on clinical specimens.

We hypothesize the correlative biomarkers will predict overall survival and six month progression free survival.

Although direct tissue analysis before and after treatment may provide the most specific biomarker predictive assays, such analyses are not likely to be accepted as conventional practice in brain tumor patients. Therefore, we propose to collect pathologic material from previous biopsy specimens from all patients whenever possible. This material will be archived for later analysis if the clinical data suggests subsets of patients have benefited from AT-101.

However, serum will be collected for analysis of apoptotic protein levels (ex bcl-2) immediately before the first dose and after one week of therapy. Protein levels measured by ELISA will be correlated with clinical outcomes including overall survival and six month progression free survival.

3.0 PATIENT ELIGIBILITY CRITERIA

3.1 Patient Sample

Sample Size:

56 patients

Accrual Rate:

Approximately 4 patients per month

Gender:
Male and Female

Age:
Patients must be at least 18 years of age.

Race:
Minorities will be actively recruited. No exclusion to this study will be based on race.

3.2 Eligibility Criteria

1. Patients must be at least 18 years of age.
2. Patients must have histologically confirmed supratentorial glioblastoma multiforme which is progressive or recurrent after radiation therapy ± chemotherapy. Patients with previously low grade glioma who progressed after radiotherapy ± chemotherapy and are biopsied and found to have glioblastoma multiforme are eligible.
3. Patients must have tumor tissue form completed and signed by a pathologist. See Section 9.5 for details.
4. Patients must have measurable contrast enhancing progressive or recurrent glioblastoma multiforme by MRI or CT imaging (Within 14 days before starting treatment).
5. Patients must have recovered from toxicity of prior therapy. An interval of at least 3 months must have elapsed since the completion of the most recent course of radiation therapy, while at least 3 weeks must have elapsed since the completion of a non-nitrosourea containing chemotherapy regimen, and at least 6 weeks since the completion of a nitrosourea containing chemotherapy regimen. NOTE: For non-cytotoxic, FDA approved agents (i.e. celebrex, thalidomide, etc.) therapy could be started 2 weeks after discontinuing this agent provided the patient has fully recovered from all toxicity associated with the agent. For investigational, non-cytotoxic agents a minimum of 3 weeks must have elasped before the patient will be eligible for this study.

6. Patients must have a Karnofsky performance status ≥ 60% (i.e. the patient must be able to care for himself/herself with occasional help from others).

7. Patients must have the following hematologic, renal and liver function. Patients must meet the following laboratory criteria: absolute neutrophil count ≥1500/mm^3; Platelets ≥ 100,000/mm^3; Creatinine ≤ 1.5mg/dl; Total Bilirubin ≤ 1.5mg/dl; Transaminases ≤ 2.5 times above the upper limits of the institutional norm

8. Patients must be able to provide written informed consent.

9. Women of childbearing potential must have a negative serum pregnancy test. The effects of AT-101 on the developing human fetus at the recommended therapeutic dose are unknown. For this reason and because Bcl-2 inhibitors have the potential to be teratogenic, women of child-bearing potential and men must agree to use adequate contraception (hormonal or barrier method of birth control; abstinence) prior to study entry and for the duration of study participation, and for at least one month following the last dose of AT-101. Should a woman become pregnant or suspect she is pregnant while participating in this study, she should inform her treating physician immediately.

10. Patients must have a Mini Mental State Exam score ≥ 15.

3.3 Ineligibility Criteria

Patients with any of the following are ineligible for this research study:

1. Patients with serious concurrent infection or medical illness which would jeopardize the ability of the patient to receive the treatment outlined in this protocol with reasonable safety. (Examples of medical illnesses are [but not limited to] the following: uncontrolled hypertension, symptomatic congestive heart failure, unstable angina pectoris, cardiac arrhythmia, or psychiatric illness/social situation that would limit compliance with study requirements.)

2. Patients who are pregnant or breast-feeding.

3. Patients who have received more than two prior treatments.

4. Patients who have been previously treated with gossypol, or have allergies to gossypol.

5. Patients receiving concurrent therapy for their tumor (i.e. chemotherapeutics or investigational agents). Concurrent steroid use is allowed.

6. Patients with a concurrent malignancy are ineligible unless they are patients with curatively treated carcinoma-in-situ or basal cell carcinoma of the skin. Patients with a prior malignancy are ineligible unless they have been free of disease for ≥ five years.

7. Patients with ≥ Grade 2 sensory neuropathy based on the NCI CTCAE.

8. Patients who are taking iron supplements.

9. Patients with any condition (*e.g.*, gastrointestinal tract disease resulting in an inability to take oral medication or a requirement for IV alimentation, prior surgical procedures affecting absorption, or active peptic ulcer disease) that impairs their ability to swallow pills are excluded.

10. Patients cannot be receiving cytochrome P450-inducing anticonvulsants (EIAEDs; *e.g.*, phenytoin, carbamazepine, phenobarbital, primidone, oxcarbazepine)

11. Patients with malabsorption syndrome, disease significantly affecting gastrointestinal function, or resection of the stomach or small bowel are excluded. Subjects with ulcerative colitis, inflammatory bowel disease, or a partial or complete small bowel obstruction are also excluded.

12. Eligibility of patients receiving any other medications or substances known to affect or with the potential to affect the activity or pharmacokinetics of AT-101 will be determined following review of their case by the Principal Investigator.

13. Patients with symptomatic hypercalcemia that is > Grade 2 (according to CTCAE).

14. Requirement for routine use of hematopoietic growth factors (including granulocyte colony stimulating factor, granulocyte macrophage colony stimulating factor, or interleukin-11) or platelet transfusions to maintain absolute neutrophil counts or platelets counts above the required thresholds for study entry.

15. HIV-positive patients on combination antiretroviral therapy are ineligible because of the potential for pharmacokinetic interactions with AT-101. In addition, these patients are at increased risk of lethal infections when treated with marrow-suppressive therapy. Appropriate studies will be undertaken in patients receiving combination antiretroviral therapy when indicated.

4.0 TREATMENT PLAN

4.1 Treatment Schema

Treatment Cycles – 28 days

Weeks 1-3: AT-101 20mg p.o. daily
Week 4: Rest, no treatment

↓

MRI performed prior to every odd treatment cycle (every 8 weeks)

Treatment for this protocol will be administered on an outpatient basis. Each patient will receive 20mg of AT-101 orally every day for 3 weeks (21 days) of each cycle. Each treatment cycle is 28 days. Tumor tissue and blood samples will be obtained from each patient prior to beginning treatment with AT-101 (See section 9.5). Treatment of an individual patient will continue until there is objective or clinical evidence of either disease progression or treatment-related, dose-limiting toxicity, or if the patient decides to discontinue treatment for any reason. All patients will be given drug to take home and a drug calendar that explicitly details how many tablets of AT-101 the patient will be taking each day. The patient will be asked to record the amount of AT-101 actually taken as well as the date and time of each dose in a pill diary.

Prior to starting each treatment cycle the patient must meet the following criteria on Day 1 of each treatment cycle:

- ANC $\geq$ 1,500/mcL
- Platelets $\geq$ 100,000/mcL
- Non-hematologic toxicity recovered to $\leq$ grade 1, tolerable grade 2 or pretherapy baseline
- No medical condition or other circumstance (including progressive disease) exists that, in the opinion of the investigator, would make the patient unsuitable for further participation in the study.

4.2 Treatment Requirements

All eligible patients who consent to this study must have a baseline pretreatment MRI or CT scan. This scan must be done up to 14 days prior to the initiation of treatment. This MRI will be used for determining tumor response. An MRI (with bi-dimensional measurement) is preferred for evaluating tumor size. However, if the patient is unable to undergo MR (because of presence of a pacemaker, aneurysm clip or claustrophobia), CT scans will be performed consistently and used for analysis.

Patient enrollment and commencement of protocol therapy may begin as soon as is deemed medically appropriate. The patient will then receive AT-101 as described and receive a clinical assessment every 28 days (4 weeks or 1 cycle), with bi-dimensional scans performed before every odd treatment cycle (i.e. prior to cycles 1,3,5 etc). If during any scheduled MRI, patient has a Complete or Partial Response, MRI should be repeated prior to next cycle. Patients will then return to the every odd cycle schedule. This is required to confirm duration of response. All patients will be followed for survival.

4.3 Drug Administration

AT-101

Treatment will be administered on an outpatient basis.

Patients should take two (2) 10-mg tablets of AT-101, once per day for 21 out of 28 days (total dose, 20 mg) at approximately the same time each day at least 1 hour before or at least 1 hour after eating food. Patients must not take drugs, foods, supplements, or over-the-counter medications containing divalent cations (e.g., dairy products, calcium supplements, or antacids) for 2 hours before to 4 hours after each dose of AT-101. If the patient vomits after taking AT-101, they should **not** retake the dose.

Patients should be instructed to keep their AT-101 tablets in the refrigerator in the original bottle and protected from light.

Patients will be monitored for cardiac function: 12-lead ECG and serum troponin level at baseline and serum troponin level alone after the last dose of AT-101 in each cycle of treatment.

4.4 Miscellaneous

Corticosteroids

A reasonable dose of corticosteroid (e.g. dexamethasone) will be determined on clinical grounds for each patient before beginning the first cycle of AT-101. An effort will be made to keep the patient on this steroid dose until the next scan is obtained. Changing steroid doses will complicate the interpretation of response. Corticosteroid doses can be tapered as clinically indicated if the patient appears to be responding to therapy as judged by serial scans.

Antiemetics

To date there is no evidence of the need for routine prophylactic use of antiemetics with AT-101. No premedication regimens are recommended for AT-101.

Hematopoietic Growth Factors

Prophylactic use of hematopoietic growth factors (G-CSF, GM-CSF, or IL-11) is not permitted during the first cycle of treatment. Their use in subsequent cycles is permissible only after consultation with the ABTC Central Office . Erythropoietin use is acceptable during all cycles of treatment per institutional guidelines.

Prohibited Medications

The following concomitant medications and therapies are NOT to be administered to patients while receiving AT-101:

Patients should avoid drugs, foods, supplements, or over-the-counter medications containing divalent cations (e.g., dairy products, calcium supplements, or antacids) for 2 hours before to 4 hours after each dose of AT-101.

5.0 DOSE MODIFICATION FOR TOXICITY

5.1 Dose Modification

In the event of toxicity, the dose of AT-101 will be adjusted according to the guidelines shown in the Dose Delays/Dose Modifications table that follows. Dose adjustments for hematologic toxicity are based on the blood counts obtained in preparation for the day of treatment.

Patients with adverse events that are manageable with supportive therapy may not require dose reductions (*e.g.*, nausea/vomiting may be treated with antiemetics, diarrhea may be treated with loperamide, electrolyte abnormalities may be corrected with supplements rather than by dose reduction).

Dose Level	AT-101 Dose
-2	Only 1 dose reduction is permitted. If a patient requires more than one dose reduction they should come off treatment.
-1	10 mg, daily<br>(1 10-mg tablet)
0	20 mg daily<br>(2 10-mg tablets)

In the event of an adverse event at least possibly related to AT-101, the doses will be adjusted according to the guidelines shown in the Dose Modifications table that follows. If an adverse event is not covered in the table, doses may be reduced or held at the discretion of the investigator for the patient's safety.

Patients who require dose delays will have their treatment delayed and will be re-evaluated when clinically appropriate. The re-evaluation interval will be based on standard institutional care criteria, the nature of the adverse event or toxicity observed, and the clinical judgment of the investigator. If the adverse event or toxicity is not resolved when re-evaluated, further delay may occur. If a treatment delay of more than 2 weeks (14 days) is required, the patient will be withdrawn from the treatment portion of the study, but will be followed to the resolution of the adverse event or toxicity. Treatment may also be delayed for logistic or personal reasons, but the treating investigator should contact the ABTC Central Office to discuss the case of any patient whose treatment has been delayed for more than 2 weeks (14 days) for such reasons, prior to resuming study therapy. The central office will allow up to a maximum of 4 weeks delay for personal reasons.

Event	AE Grade	Dose Modification
**Hematologic Adverse Events** (Grading based on blood counts obtained in preparation for day of treatment.)	Grade 0-2	No dose modification; administer 100% of dose .
	Grade 2: persistent and intolerable	Consider dose delays or dose reductions to the next lower dose level.
	Grade 3: no fever or infection	Consider dose delays or dose reductions to the next lower dose level.
	Grade 3 ANC: with fever or infection	Hold AT-101 until recovery to ANC ≥ 1.5 x 10^9/L a then reduce one dose level.[a]
	Grade 4	Skip dose and hold subsequent dosing until recovery to ANC ≥ 1.5 x 10^9/L and platelets ≥ 100 x 10^9/L then reduce one dose level.[a]
**Non-hematologic Adverse Events**	Grade 0-2	No dose modification; administer 100% of dose .
	Grade 2: persistent and intolerable	Consider dose delays or dose reductions to the next lower dose level.
	Grade 3 or 4	Hold AT-101 until recovery to ≤ grade 1, then reduce one dose level if continued therapy is medically warranted.[a]
**Elevated serum troponin level**		Hold AT-101 and send patient for cardiac evaluation. The following tests are recommended: Serial troponin testing, echocardiogram and consideration of performing trans-esophageal echocardiography (TEE) or PET scanning to exclude direct cardiac involvement by the tumor (if this is determined to be appropriate).<br><br>Consult ABTC Central Office to determine if it is in the best interest of the patient to continue in the study.

[a] Patients will be withdrawn from the study if they fail to recover to grade 0 to 1 (or to starting values for pre-existing laboratory abnormalities) or tolerable grade 2 from a treatment-related adverse event within 14 days of the planned treatment day unless the investigator and ABTC Central Office agree that the subject should remain in the study because of evidence that the patient is/may continue deriving benefit from continuing study treatment.

5.1.1 Guidelines for the Evaluation of Patients with Nausea, Vomiting, and/or Abdominal Pain

If a patient has ≥ Grade 2 nausea, ≥ Grade 2 vomiting, or any abdominal pain that lasts for longer than 48 hours, AT-101 should be held and the following actions are recommended:

1. Physical examination, including the performance of vital signs
2. Screening abdominal radiography to include an abdominal series to exclude ileus, SBO, or pneumatosis intestinalis
3. Consideration for obtaining a CT scan with contrast of the abdomen should be made based on clinical judgment

If ileus, SBO, or pneumatosis intestinalis are excluded, dosing of AT-101 TMZ may be reinstated following the provisions in the above table.

If a patient is determined to have ileus, SBO, or pneumatosis intestinalis, expectant management with supportive care is recommended, unless clinical signs or symptoms are present that suggest septicemia or abdominal catastrophe that warrants surgical management.

5.2 Major Events

Major Events are non treatment-related Grade 3 and 4 hematologic and nonhematologic toxicities. Treatment should be delayed for major events if AT-101 may further complicate the non-treatment related event. If a major event requires a delay of treatment, treatment must be delayed until toxicity is resolved (≤ Grade 2 or ≤ Baseline). If toxicity is not resolved in ≤ 2 weeks, patient will be taken off treatment. ABTC office should be consulted if you are not clear on whether to continue or delay treatment.

5.3 Toxicity Criteria

All toxicities will be graded according to the Common Terminology Criteria for Adverse Events v.4.0 beginning January 1, 2011. A copy of the CTCAE can be downloaded from the CTEP home page (http://ctep.cancer.gov). All appropriate treatment areas should have access to a copy of CTCAE version 4.0.)

5.4 Data Reporting

This study will be monitored by the Clinical Data Update System (CDUS) version 3.0. Cumulative CDUS data will be submitted quarterly to CTEP by electronic means. Reports are due January 31, April 30, July 31, and October 31.

6.0 PHARMACEUTICAL INFORMATION

6.1 AT-101

Chemical Name: R-(-)-1,1',6,6',7,7'-hexahydroxy-3,3'-dimethyl-5,5'-bis (1-methylethyl) [2,2'-Binapthalene]-8,8'-dicarboxaldehyde acetic acid

Other Names: AT-101; R-(-)-gossypol

Classification: Bcl protein inhibitor

CAS Registry Number: 90141-22-3

Molecular Formula: $C_{32}H_{34}O_{10}$ **M.W.:** 578.7 grams/mole

Mode of Action: R-(-)-gossypol acetic acid, the levorotatory enantiomer of gossypol acetic acid, is a small molecule that induces cancer cell apoptosis by inhibiting the BH3 domain of the anti-apoptotic Bcl proteins, Bcl-2 and Bcl-xL. Also an Mcl-1 inhibitor.

How Supplied: R-(-)-gossypol acetic acid is supplied by Ascenta Therapeutics, Inc. and distributed by the CTEP, DCTD, NCI. R-(-)-gossypol acetic acid is supplied as a 10 mg immediate release tablet in high density polyethylene (HDPE) opaque bottles containing 200 tablets. The inactive ingredients contained in each tablet are silicified microcrystalline cellulose, sodium starch glycolate, and stearic acid.

Storage: Store R-(-)-gossypol acetic acid in the refrigerator (2°C to 8°C) and protect from light.

Stability: Stability studies with the 10 mg dosage form are ongoing.

Route(s) **of Administration:** Oral

Method of Administration: Administer at the same time each day at least 1 hour before or after a meal. If a patient vomits after taking R-(-)-gossypol acetic acid, instruct the patient to not retake the dose. Do not crush tablets.

Patient Implications: Do not take drugs, foods, supplements, or over-the-counter medications containing divalent cations (e.g., dairy products, calcium supplements, or antacids) for 2 hours before to 4 hours after each dose of R-(-)-gossypol acetic acid.

Availability

AT-101 is an investigational agent supplied to investigators by the Division of Cancer Treatment and Diagnosis (DCTD), NCI.

AT-101 is provided to the NCI under a Collaborative Agreement between Ascenta Therapeutics, Inc. and the DCTD, NCI (see Section 13).

Agent Ordering

NCI supplied agents may be requested by the Principal Investigator (or their authorized designee) at each participating institution. Pharmaceutical Management Branch (PMB) policy requires that agent be shipped directly to the institution where the patient is to be treated. PMB does not permit the transfer of agents between institutions (unless prior approval from PMB is obtained.) The CTEP assigned protocol number must be used for ordering all CTEP supplied investigational agents. The responsible investigator at each participating institution must be registered with CTEP, DCTD through an annual submission of FDA form 1572 (Statement of Investigator), Curriculum Vitae, Supplemental Investigator Data Form (IDF), and Financial Disclosure Form (FDF). If there are several participating investigators at one institution, CTEP supplied investigational agents for the study should be ordered under the name of one lead investigator at that institution.

Agent may be requested by completing a Clinical Drug Request (NIH-986) and mailing it to the Pharmaceutical Management Branch, DCTD, NCI, 9000 Rockville Pike, EPN Room 7149, Bethesda, MD 20892-7422 or faxing it to (301) 480-4612. For questions call (301) 496-5725.

Agent Accountability

Agent Inventory Records – The investigator, or a responsible party designated by the investigator, must maintain a careful record of the inventory and disposition of all agents received from DCTD using the NCI Drug Accountability Record Form (DARF). (See the CTEP home page at http://ctep.cancer.gov for the Procedures for Drug Accountability and Storage and to obtain a copy of the DARF and Clinical Drug Request form.)

7.0 PROCEDURES FOR PATIENT ENTRY ON STUDY

The ABTC ID Request and Patient Registration Form for this protocol, and the signed consent should be FAXed to the ABTC Central Office. The ABTC Central Office will fax to site the completed ID Request (ID# assigned) which will confirm patient's registration. **No patient may begin treatment until this confirmation(form) is received at the registering site. NOTE:** Confirmation of the IRB approval will be required prior to patient registration

Serena Desideri, M.D.
ABTC Central Office
Tel 410 614-4400
Fax 410 614-9335

8.0 RESPONSE ASSESSMENT

8.1 Criteria for Response Assessment

Standardized response criteria are outlined below. MRI/CT scans and neurologic examinations will be used to determine the response to therapy.

Complete Response: Complete disappearance of all tumor on MRI/CT scan, off all glucocorticoids with a stable or improving neurologic examination for a minimum of 4 weeks.

Partial Response: Greater than or equal to 50% reduction in tumor size on bi-dimensional MRI/CT scan, on a stable or decreasing dose of glucocorticoids, with a stable or improving neurologic examination for a minimum of 4 weeks.

Progressive Disease: Progressive neurologic abnormalities not explained by causes unrelated to tumor progression (e.g. anticonvulsant or corticosteroid toxicity, electrolyte abnormalities, hyperglycemia, etc.) or a greater than 25% increase in the size of the tumor by MRI/CT scan. If neurologic status deteriorates, on a stable or increasing dose of steroids, or if new lesions appear on serial MRI/CT, further study treatment will be discontinued.

Stable Disease: A patient whose clinical status and MRI/CT measurements do not meet the criteria for *Complete Response*, *Partial Response or Progressive Disease.*

Note: Clinical and radiographic evidence of increased enhancement and/or brain edema may be attributed to either an anti-tumor response induced by the treatment or to tumor progression. In some instances, it may be apparent based on PET or MR spectroscopy that the increasing mass effect reflects tumor progression, but in other instances this may be uncertain, and in such cases, stereotactic or open biopsy of the intracranial tumor site may be indicated to assess treatment options. If a response against the tumor is the primary cause for the increasing mass effect, then the patient would be able to continue treatment as part of this protocol as long as they are able to resume protocol therapy within 4 weeks of last dose. Conversely, if the edema is caused by tumor progression, the participant would be "off treatment" and would be offered other treatment options.

8.2 Assessment of Response

Timing of the Evaluation of Response to AT-101 : Assessment of response to AT-101 (i.e. determining whether the patient will receive additional cycles of AT-101 after the second treatment cycle) will begin with the MRI/CT (Bi-dimensional Imaging) performed just prior to *every odd* cycle of AT-101 (within -5 days of starting cycle). All scans are to be compared to the smallest measurement scan to date. Patients will be classified as responders if they have a minimum duration of response for 4 weeks at any time after the first treatment cycle of AT-101. If at any scheduled exam, patient has a complete or partial response, a scan must be repeated prior to the next cycle (even cycle), then return to the every odd cycle schedule. This assessment is needed to determine actual duration of response.

8.3 Quality Assurance

The Study chairmen will be reviewing the records in detail for the following:

Neuropathology: The neuropathologic diagnosis of glioblastoma multiforme should be made at the respective institution. If any question arises regarding the accuracy of the neuropathologic diagnosis, slides (and pathological blocks, if necessary) will be reviewed by the central review pathologist. All patients with a documented complete or partial response will have representative pathology slides undergo central review.

Neuroradiology: MRI/CT (Bi-dimensional Imaging) scans of patients showing tumor response will be centrally reviewed by a neuroradiologist who will independently assess tumor size and compute percent tumor regression.

Neuro-oncology: The local investigator at the participating institution will communicate to the protocol chairman any unexpected neurological effects such as change in seizure frequency, alteration in neuromuscular function, alteration in cognitive function, or fluctuations in serum anticonvulsant drug levels.

Adherence to protocol therapy: As a quality assurance measure for the treatment delivered on this protocol, primary patient records may be reviewed. The records to be examined will be selected retrospectively and at random; complete records must therefore be maintained on each patient treated on the protocol. These records should include primary documentation (e.g., laboratory report slips, X-ray reports, scan reports, pathology reports, physician notes, etc.), which confirm that:

- The patient met each eligibility criterion.
- Signed informed consent was obtained prior to treatment.
- Treatment was given according to protocol (dated notes about doses given; any reasons for any dose modifications).
- Toxicity was assessed according to protocol (laboratory report slips, etc.).
- Response was assessed according to protocol (MRI/CT scan, lab reports, dated notes on measurements and clinical assessment, as appropriate).
- NCI Drug Accountability Records were maintained for this protocol.

9.0 MONITORING OF PATIENTS

9.1 Table of Required Observations

	Baseline	Cycle 1, week 2	Pre Odd cycles	Pre Even Cycles	Off Treatment (w/in 7 days of last dose)
AE Evaluation			4	4	7,12
MRI	1		11		13
H&P,Neuro exam	1		11	11	7
Minimental	1				
KPS	1		11		7
Vital Signs	1,5,6		11,6	11,6	6,7
Hematology	1,9		4,8,9	4,8,9	7,9
Serum Chemistry	1,10		4,10	4,10	7,10
LDH level	1		4	4	7
Serum Troponin Level	1		2,14	2,14	15
Serum Pregnancy Test	1				
Laboratory correlative tissue sample	1,3				
12-lead ECG	1				
Serum Biomarkers	16	16			

1 – All baseline measurements must be done within 14 calendar days prior to start of treatment administration unless otherwise specified.
2 – See section 5.1 for guidelines for elevated serum troponin.
3 – Archived Tissue to be obtained and sent unstained paraffin-embedded tissue (from the most recent tumor block available). Investigators should make efforts to collect and ship one block or 15-20 slides. See Section 9.5 for details.
4 – within -3 days
5 – Height should be obtained at baseline
6 – Vital signs include temperature, blood pressure, respiratory rate, and heart rate (weight only needs to be obtained every odd cycle)
7 – Repeat only if > 3 calendar days since last eval/test
8 – If ANC < 1500 or plts < 100,000, CBCs/differentials will be repeated twice a week until counts are recovered (ANC ≥1500 or plts ≥100,000) per protocol. If counts are recovered (ANC ≥1500 or plts ≥100,000) on day of scheduled drawing do not repeat till next protocol schedule day.
9 – CBC with differential count and platelet count
10 – Na, K, HCO3, Ca, glucose, BUN, creatinine, total bilirubin, SGOT, SGPT, alkaline phosphatase, albumin, GGT, total protein
11 – within -5 calendar days (MRI should be obtained and evaluated prior to start of new cycle)
12 – Collect for at least 30 days post last dose of AT-101. Patients who are discontinued due to an AE will be followed until AE resolves.
13 – repeat only if > 14 days since date of last scan
14 – Needs to be drawn after the last dose of AT-101 in each treatment cycle
15 – Needs to be drawn the last day of treatment
16 – Serum Biomarkers are being collected within 24 hours before the first dose of AT-101 and after one week of therapy (any time during week 2 of cycle 1). See Section 9.5.2 for details.

9.2 Adverse Event Definition and Reporting

Patients will be evaluated for toxicity if they have received at least one dose of AT-101.

9.2.1 Definition - Adverse Event

Adverse event is defined as any unfavorable and unintended sign (including an abnormal laboratory finding), symptom or disease temporally associated with the use of a medical treatment or procedure regardless of whether it is considered related to the medical treatment or procedure (attribution of unrelated, unlikely, possible, probable, or definite).

9.2.2 Recording of Adverse Events - ABTC AE Form

- **Document on ABTC Adverse Event Form**
- **Using AdEERS Application**

1) **CTCAE term (AE description) and grade:** The descriptions and grading scales found in the revised NCI Common Terminology Criteria for Adverse Events (CTCAE) version 4.0 will be utilized for AE reporting beginning January 1, 2011. All appropriate treatment areas should have access to a copy of the CTCAE version 4.0. A copy of the CTCAE version 4.0 can be downloaded from the CTEP web site (http://ctep.cancer.gov).

2) The Investigator will monitor each patient closely for the development of adverse events and toxicities and record all such events on the ABTC AE Form. Each single sign or symptom must be reported separately. The DCTD/NCI Common Terminology Criteria for Adverse Events, CTCAE v.4.0 will be employed to record these toxicities beginning January 1, 2011. **A copy of the CTCAE version 4.0 can be downloaded from the CTEP home page (http://ctep.cancer.gov). All appropriate treatment areas should have access to a copy of the CTCAE version 4.0. You must use one of the CTCAE criteria to define your event.**

3) Adverse events not included in the CTCAE v.4.0 should be reported and graded under the Other adverse event within the appropriate category and grade 1 to 5 according to the general grade definitions, mild, moderate, severe, life-threatening, fatal or disabling, as provided in the CTCAE or the CTCAE Manual. For new adverse events, the investigator may use the Adverse Event Module, Appendix I of the CTCAE, v.4.0. Also, new adverse events may be submitted to the CTEP Help Desk for annual evaluation by the CTCAE Change Management Committee.

4) Abnormal lab results which are graded by NCI CTCAE v.4.0 will be recorded on ABTC Laboratory Checklist, along with documented attribution, not on AE form. However, if an action was conducted due to this abnormality (e.g. RBC transfusion due to low Hgb) this would be recorded on the AE form also.

All adverse events should be followed up in accordance with good medical practice. Abnormalities of laboratory events which, in the opinion of the Investigator, constitute adverse events (even if not serious) should be followed.

9.2.3 Relationship

The Investigator must document his/her opinion of the relationship of the event to study medication as follows:

- *Unrelated*
  The adverse event is clearly not related to the investigational agent(s).
- *Unlikely*
  The adverse event is doubtfully related to the investigational agent(s).
- *Possible*
  The adverse event may be related to the investigational agent(s).
- *Probable*
  The adverse event is most likely related to the investigational agent(s).
- *Definite*
  The adverse event is clearly related to the investigational agent(s).

9.3 Comprehensive Adverse Events and Potential Risks Lists (CAEPRs)

Comprehensive Adverse Events and Potential Risks list (CAEPR)
for
(R)-(-)-Gossypol acetic acid (AT-101) (NSC 726190)

The Comprehensive Adverse Event and Potential Risks list (CAEPR) provides a single list of reported and/or potential adverse events (AE) associated with an agent using a uniform presentation of events by body system. In addition to the comprehensive list, a subset, the Agent Specific Adverse Event List (ASAEL), appears in a separate column and is identified with ***bold*** and ***italicized*** text. This subset of AEs (ASAEL) contains events that are considered 'expected' for expedited reporting purposes only. Refer to the 'CTEP, NCI Guidelines: Adverse Event Reporting Requirements' http://ctep.info.nih.gov/protocolDevelopment/default.htm#adverse_events_adeers for further clarification. *Frequency is provided based on 230 patients.* Below is the CAEPR for (R)-(-)-Gossypol acetic acid (AT-101).

Version 2.1, January 6, 2010

Adverse Events with Possible Relationship to (R)-(-)-Gossypol acetic acid (AT-101) (CTCAE 4.0 Term) [n= 230]			EXPECTED AEs FOR ADEERS REPORTING<br><br>Agent Specific Adverse Event List (ASAEL)
**Likely (>20%)**	**Less Likely (<=20%)**	**Rare but Serious (<3%)**	***Expected***
BLOOD AND LYMPHATIC SYSTEM DISORDERS			
	Anemia		
GASTROINTESTINAL DISORDERS			
	Abdominal distension		***Abdominal distension***
Abdominal pain			***Abdominal pain***
	Constipation		***Constipation***
Diarrhea			***Diarrhea***
	Dry mouth		***Dry mouth***
	Dyspepsia		
	Gastritis		
	Gastrointestinal disorders - Other (pneumatosis intestinalis)		***Gastrointestinal disorders - Other (pneumatosis intestinalis)***
	Ileus		***Ileus***
Nausea			***Nausea***
	Small intestinal obstruction		***Small intestinal obstruction***
Vomiting			***Vomiting***
GENERAL DISORDERS AND ADMINISTRATION SITE CONDITIONS			
	Edema limbs		
Fatigue			***Fatigue***
	Fever		
INFECTIONS AND INFESTATIONS			
	Infections and infestations – Other (Infection – Select)		***Infections and infestations – Other (Infection – Select)***
INVESTIGATIONS			
	Alanine aminotransferase increased		***Alanine aminotransferase increased***
	Alkaline phosphatase increased		
	Aspartate aminotransferase increased		***Aspartate aminotransferase increased***

	Blood bilirubin increased		
	Cardiac troponin I increased		
	Cardiac troponin T increased		
	Creatinine increased		
	Weight loss		***Weight loss***
	White blood cell decreased		
METABOLISM AND NUTRITION DISORDERS			
Anorexia			***Anorexia***
	Dehydration		***Dehydration***
	Hypocalcemia		
	Hypokalemia		***Hypokalemia***
	Hypomagnesemia		***Hypomagnesemia***
MUSCULOSKELETAL AND CONNECTIVE TISSUE DISORDERS			
	Back pain		
	Pain in extremity		
NERVOUS SYSTEM DISORDERS			
	Dizziness		***Dizziness***
	Dysgeusia		***Dysgeusia***
	Headache		
PSYCHIATRIC DISORDERS			
	Insomnia		
	Libido decreased		***Libido decreased***
REPRODUCTIVE SYSTEM AND BREAST DISORDERS			
	Azoospermia		***Azoospermia***
	Irregular menstruation		***Irregular menstruation***
RESPIRATORY, THORACIC AND MEDIASTINAL DISORDERS			
	Cough		
	Dyspnea		***Dyspnea***
SKIN AND SUBCUTANEOUS TISSUE DISORDERS			
	Dry skin		***Dry skin***
	Pruritus		
	Rash maculo-papular		

[1] This table will be updated as the toxicity profile of the agent is revised. Updates will be distributed to all Principal Investigators at the time of revision. The current version can be obtained by contacting PIO@CTEP.NCI.NIH.GOV. Your name, the name of the investigator, the protocol and the agent should be included in the e-mail.

Also reported on (R)-(-)-Gossypol acetic acid (AT-101) trials but with the relationship to (R)-(-)-Gossypol acetic acid (AT-101) still undetermined:

BLOOD AND LYMPHATIC SYSTEM DISORDERS - Lymph node pain
CARDIAC DISORDERS - Myocardial infarction; Pericardial effusion; Sinus tachycardia
EYE DISORDERS - Blurred vision; Dry eye
GASTROINTESTINAL DISORDERS - Dysphagia; Flatulence; Hemorrhoids; Mucositis oral
GENERAL DISORDERS AND ADMINISTRATION SITE CONDITIONS - Chills; Edema face
INFECTIONS AND INFESTATIONS - Infections and infestations - Other (acute bacterial pancreatitis); Infections and infestations - Other (Opportunistic infection associated with >= Grade 2 Lymphopenia)
INJURY, POISONING AND PROCEDURAL COMPLICATIONS - Bruising
INVESTIGATIONS - Neutrophil count decreased; Platelet count decreased
METABOLISM AND NUTRITION DISORDERS - Hyperglycemia; Hyperuricemia; Hyponatremia
MUSCULOSKELETAL AND CONNECTIVE TISSUE DISORDERS - Arthralgia; Bone pain; Myalgia
NERVOUS SYSTEM DISORDERS - Depressed level of consciousness; Olfactory nerve disorder
PSYCHIATRIC DISORDERS - Anxiety

RENAL AND URINARY DISORDERS - Acute kidney injury; Hematuria; Proteinuria; Renal and urinary disorders - Other (urine odor abnormal)
REPRODUCTIVE SYSTEM AND BREAST DISORDERS - Testicular pain
RESPIRATORY, THORACIC AND MEDIASTINAL DISORDERS - Allergic rhinitis; Epistaxis; Pleural effusion; Pleuritic pain
SKIN AND SUBCUTANEOUS TISSUE DISORDERS - Alopecia
VASCULAR DISORDERS - Hot flashes; Thromboembolic event

Note: (R)-(-)-Gossypol acetic acid (AT-101) in combination with other agents could cause an exacerbation of any adverse event currently known to be caused by the other agent, or the combination may result in events never previously associated with either agent.

9.4 Expedited Adverse Event Reporting

Adverse Event Expedited Reporting System - AdEERS

Expedited AE reporting for this study must use AdEERS (Adverse Event Expedited Reporting System), accessed via the CTEP home page (http://ctep.cancer.gov). The reporting procedures to be followed are presented in the "CTEP, NCI Guidelines: Adverse Event Reporting Requirements" which can be downloaded from the CTEP home page (http://ctep.cancer.gov). These requirements are briefly outlined in the table below (Section 9.4.1).

In the rare occurrence when Internet connectivity is disrupted, a 24-hour notification is to be made to CTEP by telephone at 301-897-7497. An electronic report must be submitted immediately upon re-establishment of internet connection. Please note that all paper AdEERS forms have been removed from the CTEP website and can no longer be accepted.

'Expectedness': AEs can be 'Unexpected' or 'Expected' for expedited reporting purposes only. 'Expected' AEs (the ASAEL) are ***bold and italicized*** in the CAEPR.

9.4.1 Expedited Reporting Guidelines

AdEERS Reporting Requirements for Adverse Events that occur within 30 Days[1] of the Last Dose of the Investigational Agent on Phase 2 and 3 Trials

Phase 2 and 3 Trials									
	**Grade 1**	**Grade 2**	**Grade 2**	**Grade 3**		**Grade 3**		**Grades 4 & 5[2]**	**Grades 4 & 5[2]**
	**Unexpected and Expected**	**Unexpected**	**Expected**	**Unexpected**		**Expected**		**Unexpected**	**Expected**
				**with Hospitali-zation**	**without Hospitali-zation**	**with Hospitali-zation**	**without Hospitali-zation**		
**Unrelated Unlikely**	Not Required	Not Required	Not Required	10 Calendar Days	Not Required	10 Calendar Days	Not Required	10 Calendar Days	10 Calendar Days
**Possible Probable Definite**	Not Required	10 Calendar Days	Not Required	10 Calendar Days	10 Calendar Days	10 Calendar Days	Not Required	24-Hour; 5 Calendar Days	10 Calendar Days

1 **Adverse events with attribution of possible, probable, or definite that occur greater than 30 days after the last dose of treatment with an agent under a CTEP IND require reporting as follows:**
AdEERS 24-hour notification followed by complete report within 5 calendar days for:
- Grade 4 and Grade 5 unexpected events

AdEERS 10 calendar day report:
- Grade 3 unexpected events with hospitalization or prolongation of hospitalization
- Grade 5 expected events

2 Although an AdEERS 24-hour notification is not required for death clearly related to progressive disease, a full report is required as outlined in the table.

December 15, 2004

Note: All deaths on study require both routine and expedited reporting regardless of causality. Attribution to treatment or other cause should be provided.

- Expedited AE reporting timelines defined:
  - "24 hours; 5 calendar days" – The investigator must initially report the AE via AdEERS within 24 hours of learning of the event followed by a complete AdEERS report within 5 calendar days of the initial 24-hour report.
  - "10 calendar days" - A complete AdEERS report on the AE must be submitted within 10 calendar days of the investigator learning of the event.

- Any medical event equivalent to CTCAE grade 3, 4, or 5 that precipitates hospitalization (or prolongation of existing hospitalization) must be reported regardless of attribution and designation as expected or unexpected with the exception of any events identified as protocol-specific expedited adverse event reporting exclusions.

- Any event that results in persistent or significant disabilities/incapacities, congenital anomalies, or birth defects must be reported via AdEERS if the event occurs following treatment with an agent under a CTEP IND.

- Use the NCI protocol number and the protocol-specific patient ID assigned during trial registration on all reports.

9.4.2 Exceptions to Expedited Reporting Guidelines

CTCAE Category	Adverse Event	Grade	Hospitalization/ Prolongation of Hospitalization	Attribution	Comments
BLOOD/BONE MARROW	Hemoglobin	2	No	Possible	
	Leukoctyes	2-4	No	Possible	
	Platelets	2	No	Possible	
Gastrointestinal	Diarrhea	3	No	Possible	
	Nausea	3	No	Possible	
	Vomiting	3	No	Possible	

9.4.3 ABTC Reporting

Those Adverse Events described in Section 9.4.1 requiring immediate reporting (within 24 hours) to the NCI Investigational Drug Branch, CTEP will utilize the AdEERS 24-hour notification pathway accessible via web at http://ctep.cancer.gov/reporting/adeers.html).

Additionally a phone call must be made to:

Serena Desideri, MD
ABTC Data Coordinator
Office: (410) 614-4400
Fax: (410) 614-9335
Pager: 410-283-7752

In addition to the initial 24-hour report, all Adverse Events must be documented on the ABTC AE Form and using the AdEERS application. The AdEERS Form should be submitted to the ABTC Central Office and CTEP/NCI within 10 days of the event. Criteria for documenting the relationship to study drug and severity will be the same as those previously described.

ABTC FAX # 410-614-9335

These events also must be reported by the Investigator to the appropriate Institutional Review Board (IRB).

Patients who are removed from study due to adverse events should be followed until the adverse event has resolved or stabilized. Copies of relevant documentation, such as laboratory reports, should be kept with the patient's study records.

9.5 Investigational Studies/Laboratory Correlates

9.5.1 Tumor Tissue

Tumor tissue will also be collected to analyze certain biomarkers. At the time of registration, prior to beginning treatment with AT-101 a tumor tissue form will need to be completed and signed by a pathologist. This form provides written documentation of the availability of tissue for this study and the pathologist's agreement to send it as described below.

Archived tumor tissue samples (tumor should be from the most recent tumor block available) will be collected, if available, for patients enrolled on this study. Investigators are to collect and ship one block or 15-20 slides of unstained, paraffin-embedded tissue. Tissue will be used for biomarker studies to determine intra-tumoral expression levels of relevant biomarkers, which may include, but are not limited to:

- Bcl-2 family protein expression (e.g., Bcl-2, Bcl-xL, MCl-1, Bax, Bak, and BH3 domain for BH3 members only);
- MGMT gene methylation status;

Every effort will be made to collect tissue from additional surgeries the patient may have while on study.

9.5.2 Serum Biomarkers

Serum will be collected from all patients for analysis of apoptotic protein levels (ex bcl-2) immediately (within 24 hours) before the first dose and after one week of therapy (any time during week 2 of cycle 1). Protein levels measured by ELISA will be correlated with clinical outcomes including overall survival and six month progression free survival.

Blood samples should be 10 ml collected in a standard non-additive collection tube (red top). After coagulation the sera should be collected and stored at -20°C until shipping. Specimens should be labeled with the date and time of collection and ABTC patient number. Samples should be shipped under dry ice to:

Keven Raisch, Ph.D.
UAB Department of Radiation Oncology
1824 6th Avenue South
Wallace Tumor Institute 686
Birmingham, AL 35294-6852
205-975-0224 (voice)
205-975-6161 (fax)

10.0 OFF TREATMENT/OFF STUDY CRITERIA

Each subject has the right to withdraw from the study at any time without prejudice. The investigator may discontinue any subject's participation for any reason, including adverse event or failure to comply with the protocol (as judged by the investigator such as compliance below 80%, failure to maintain appointments, etc.).

Should a subject withdraw from the study, the reason(s) must be stated on the case report form, and a final evaluation of the subject should be performed. (See Section 9.2)

OFF TREATMENT CRITERIA

1. Progression of Disease: Remove patient from protocol therapy at the time progressive disease is documented.
   Disease progression is defined as: Progressive neurologic abnormalities not explained by causes unrelated to tumor progression (e.g. anticonvulsant or corticosteroid toxicity, electrolyte abnormalities, hyperglycemia, etc.) or a greater than 25% increase in the measurement of the tumor by MRI/CT scan. If neurologic status deteriorates, on a stable or increasing dose of steroids, or if new lesions appear on serial MRI/CT, further study treatment will be discontinued.
2. Extraordinary Medical Circumstance: If at any time the treating physician feels constraints of this protocol are detrimental to the patient's health, remove the patient from protocol therapy.
3. Patient's refusal to continue treatment: In this event, document the reason(s) for withdrawal.
4. Failure to comply with protocol (as judged by the investigator such as compliance below 80%, failure to maintain appointments, etc.).
5. Patients who experience unacceptable toxicity, including patients who would require a 2nd dose reduction.
6. Delay in treatment > 14 days.

OFF STUDY CRITERIA

1) Patients will only be off study at time of death. (All patients will be followed every 2 months for survival.)

11.0 STATISTICAL CONSIDERATIONS

11.1 Overall Objective

The overall objective of this study is to assess the safety and efficacy of the R-(-)-gossypol (AT-101) in treating adult patients with recurrent glioblastoma multiforme (GBM). The mechanism of the AT-101 is to inhibit the heterodimerization of Bcl-2, Bcl-xL, Bcl-W, and Mcl-1 with pro-apoptotic members of the Bcl-2 family at submicromolar affinity (Investigator's Brochure, 2006). The overall survival is considered the most informative measurement to estimate the clinical benefit regarding to this treatment. Currently, there is rarely any effective treatment existing for this group of patients.

11.2 Primary Objective

To estimate an overall survival, the primary objective of the study is to estimate the overall failure rate compared to the failure rate of the NABTT historical phase II clinical trial database in the same patient population. There are 62 GBM patients who participated in three closed NABTT phase II clinical trials and were treated with single or combination of experimental chemotherapies (protocol number: 9502, 9506, 9706, 9711, and 2111). The estimated historical hazard rate is 1.67 per person-year of follow-up (95% CI: 1.3, 2.14) and an estimates historical median time of survival is 5 months.

The primary endpoint is death. The survival time is defined from time of first day of the treatment to death occurrence. The overall failure rate is expressed as hazard of failure per person-year of follow-up (the number of deaths divided by the total exposure time in the study cohort). The total patient population of this study is defined as all patients who have met eligibility criteria, not met ineligibility criteria and signed informed consent, which will be included in the statistical estimations.

To estimate the overall failure rate with sufficient precision, a total of 52 events are required from 56 patients at a accrual rate of 4 patients per month for 14 months, and with 1.5 years of additional follow-up (total study duration is 2.7 years). We assume that patients in the study will have an overall failure rate of 1.25 per person-year of follow-up, about 25% reduction compared to a hazard rate of 1.67 shown in the relevant NABTT historical database. The 52 events among the 56 patients during the planned follow-up will yield 80% power to detect an observed hazard ratio of 0.75 (1.25 vs. 1.67) to be statistically significant at an alpha level of 0.1 (one-side, A Methodologic Perspective, Piantadosi 1997). The 25% reduction in hazard rate translates into a 33% increase in median time of survival (from 5 month to 6.65 month). Moreover, the total of 52 events will yield 90% power to detect a 30% reduction in observed hazard ratio of 0.7 (1.17 vs 1.67) to be statistically significant, which increases the median time of survival in 40% (from 5 month to 7 month).

The overall failure rate will be estimated along with 95% confidence intervals. A median time of survival will be estimated using standard methods.

11.3 Secondary Objectives

1. To assess the acute and late toxicities associated with the treatment. The proportion of patients with serious or life threatening toxicities will be estimated along with 95% confidence intervals. The frequency of different type toxicities will be tabulated by each cycle of the treatment.

2. To estimate tumor response rate. The probability of tumor response (definition: section 8.1) will be estimated using binomial distribution along with 95% CI.

3. To estimate a 6-month progression-free survival rate. The 6-month progression-free survival is defined as patient who is alive and disease progression free at the time of 26-week (6 months) from first day of the treatment (definition of disease progression: section 10.0). The probability of 6-month progression-free survival will be estimated using binomial distribution.

4. Correlative study: Apoptosis can be triggered through either the intrinsic (mitochrondrial) or extrinsic (receptor) pathways. The study will focus on regulators of both the intrinsic (Bcl-2, Bcl-XL, Mcl-1, Bax, Bak) and extrinsic (FLIP, survivin, XIAP) pathways. Serum will be collected for analysis of apoptotic protein levels immediately before the first dose and after one week of therapy. Every effort will be made to collect tumor tissue before and after treatment. Descriptive statistics will be used to summarize the protein expressions and patient's MGMT methylation status.

   The Cox's proportional hazard model will be used to explore the association between change of the proteins and patient's survival status.

11.4 Interim Monitoring

This is a single arm, open label, single agent, and fixed dose phase II clinical trial. One planned safety assessment will be performed after the first 25 patients are accrued into the study and have had at least one cycle of the treatment. If the following pertain, the study will be suspended pending review by the ABTC DSMC: lower 95% confidence bound on the role of toxicities grade ≥3 (this translates to dose limiting toxicity events of any type) exceeds 18%/ or if 4 or less patients (=<16%) achieved disease progression-free survival at 6 months.

Progression-free survival at 6 months has not been proven as a validated survival surrogate. The study uses PFS-6m to describe the nature process of the disease without any effective treatment, which the PFS-6m is about 15% in this patient population. A consensus decision by ABTC, CTEP, and the sponsor is required for stopping the trial earlier, if it is necessary due to any reasons.

11.5 Decision rule

A decision rule will be implemented following the calculation of the failure rate and confidence limits to decide whether the treatment should be warrant for a future comparative trial. A 30% increasing in the median time of survival (or 26% reduction in the failure rate) compared to NABTT historical data would be considered strong evidence in favor of proceeding with a formal comparative trial. Less than 30% improvement in the median time of survival or less than 26% reduction in the failure rate compared to the historical data would be considered neither reliable nor promising enough clinically to pursue a randomized trial. However, these decision rules ultimately are partially subjective and depend greatly on the toxicities associated with the treatment.

12.0 RECORDS TO BE KEPT

Data should be faxed (410) 614-9335 or mailed to Central Office: Johns Hopkins; CRB II, Rm 1M16; 1550 Orleans Street Baltimore MD, 21231

- All data are due within 14 days of evaluation time point. Please see section 9.1 for evaluation time points.
- Serious Adverse Events, PHONE IMMEDIATELY, SEE SECTION 9.4.2

13.0 CTA

The AT-101 supplied by CTEP, DCTD, NCI used in this protocol is provided to the NCI under a Collaborative Agreement (CTA) between the Ascenta Therapeutics (hereinafter referred to as a Collaborator(s)) and the NCI Division of Cancer Treatment and Diagnosis. Therefore, the following obligations/guidelines, in addition to the provisions in the Intellectual Property Option to Collaborator(s) contained within the terms of award, apply to the use of the Agent(s) in this study:

1. Agent(s) may not be used for any purpose outside the scope of this protocol, nor can Agent(s) be transferred or licensed to any party not participating in the clinical study. Collaborator(s) data for Agent(s) are confidential and proprietary to Collaborator(s) and shall be maintained as such by the investigators. The protocol documents for studies utilizing investigational Agents contain confidential information and should not be shared or distributed without the permission of the NCI. If a copy of this protocol is requested by a patient or patient's family member participating on the study, the individual should sign a confidentiality agreement. A suitable model agreement can be downloaded from: http://ctep.cancer.gov

2. For a clinical protocol where there is an investigational Agent used in combination with (an)other investigational Agent(s), each the subject of different collaborative agreements , the access to and use of data by each Collaborator shall be as follows (data pertaining to such combination use shall hereinafter be referred to as "Multi-Party Data"):

   a. NCI must provide all Collaborators with prior written notice regarding the existence and nature of any agreements governing their collaboration with NIH, the design of the proposed combination protocol, and the existence of any obligations that would tend to restrict NCI's participation in the proposed combination protocol.

   b. Each Collaborator shall agree to permit use of the Multi-Party Data from the clinical trial by any other Collaborator solely to the extent necessary to allow said other Collaborator to develop, obtain regulatory approval or commercialize its own investigational Agent.

   c. Any Collaborator having the right to use the Multi-Party Data from these trials must agree in writing prior to the commencement of the trials that it will use the Multi-Party Data solely for development, regulatory approval, and commercialization of its own investigational Agent.

3. Clinical Trial Data and Results and Raw Data developed under a Collaborative Agreement will be made available exclusively to Collaborator(s), the NCI, and the FDA, as appropriate and unless additional disclosure is required by law or court order. Additionally, all Clinical Data and Results and Raw Data will be collected, used and disclosed consistent with all applicable federal statutes and regulations for the protection of human subjects, including, if applicable, the Standards for Privacy of Individually Identifiable Health Information set forth in 45 C.F.R. Part 164.

4. When a Collaborator wishes to initiate a data request, the request should first be sent to the NCI, who will then notify the appropriate investigators (Group Chair for Cooperative Group studies, or PI for other studies) of Collaborator's wish to contact them.

5. Any data provided to Collaborator(s) for Phase 3 studies must be in accordance with the guidelines and policies of the responsible Data Monitoring Committee (DMC), if there is a DMC for this clinical trial.

6. Any manuscripts reporting the results of this clinical trial must be provided to CTEP for immediate delivery to Collaborator(s) for advisory review and comment prior to submission for publication. Collaborator(s) will have 30 days from the date of receipt for review. Collaborator shall have the right to request that publication be delayed for up to an additional 30 days in order to ensure that Collaborator's confidential and proprietary data, in addition to Collaborator(s)'s intellectual property rights, are protected. Copies of abstracts must be provided to CTEP for forwarding to Collaborator(s) for courtesy review as soon as possible and preferably at least three (3) days prior to submission, but in any case, prior to presentation at the meeting or publication in the proceedings. Press releases and other media presentations must also be forwarded to CTEP prior to release. Copies of any manuscript, abstract and/or press release/ media presentation should be sent to:

   Regulatory Affairs Branch, CTEP, DCTD, NCI
   Executive Plaza North, Suite 7111
   Bethesda, Maryland 20892
   FAX 301-402-1584
   Email: anshers@ctep.nci.nih.gov

   The Regulatory Affairs Branch will then distribute them to Collaborator(s). No publication, manuscript or other form of public disclosure shall contain any of Collaborators confidential/ proprietary information.

14.0 REFERENCES

Bushunow P., M. Reidenberg, J. Winfield, *et al*. (1999). Gossypol treatment of recurrent adult malignant gliomas. *Journal of Neuro-oncology 43:79-86.*. Vol. 14 A282.

Flack, M.R., R.G. Pyle, N.M. Mullen, *et al*. (1993). Oral gossypol in the treatment of metastatic adrenal cancer. *J Clin Endocrinol Metab* 76:1019-1024.

Fuh, B.R., C-L. Chen, S. Wang, *et al*. (2006). (-)-Gossypol inhibits cell proliferation and induces apoptosis in rhabdomyosarcoma *in vitro*. *Proc Am Assoc Cancer Res*. 47:A4727.

Heiser, D., V. Labi, M. Erlacher, A. Villunger. (2004). The Bcl-2 protein family and its role in the development of neoplastic disease. *Exp. Gerontol*. 39:1125-1135.

Heywood, R. (1988). The toxicology of gossypol acetic acid and (-)-gossypol. *Contraception* 37:185-190.

Investigator's Brochure (2006). AT-101 [R-(-)-gossypol acetic acid]. Ascenta Therapeutics, Inc. Version 2. Version Date: March 31, 2006.

James, D.F., C.E. Prada, and J.E. Castro. (2005). AT 101, an inhibitor of Bcl-2 family members, is cytotoxic to a heterogeneous group of CLL samples and synergistic with rituximab. *Blood*. 106(11):A2979.

Kumar, S., M. Kline, T. Kimlinger, *et al*. (2005). AT-101, a small molecule inhibitor of Bcl-2 family proteins, has significant *in vitro* activity in multiple myeloma. *Blood* 106(11):A1581.

Letai, A. (2003). BH3 domains as BCL-2 inhibitors: prototype cancer therapeutics. *Expert Opin Biol Ther*. 3(2):293-304.

Paoluzzi L., M. Gonen, L. Toner, *et al*. (2005). Targeting antiapoptotic Bcl-2 family members with AT-101 in pre-clinical models of aggressive lymphoma in combination with cyclophosphamide (C) and rituximab (R) produces a marked improvement in therapeutic efficacy. *Blood*. 106(11):A2420.

Prada, C.E., J.E. Castro, D. Zhai, *et al*. (2005). R-(-)-gossypol (AT101) binds to Bcl-2 family proteins and induces apoptosis in CLL. *Blood*. 106(11):A2978.

Saleh, M., H. Pitot, J. Hartung, *et al*. (2005). Phase I trial of AT-101, an orally bioavailable inhibitor of Bcl-2, in patients with advanced malignancies. AACR-NCI-EORTC International Conference on Molecular Targets and Cancer Therapeutics, November 2005. Abstract: C89.

Sang, G.W., Y.G. Zhang, Q.X. Shi, *et al*. (1980). [Chronic toxicity of gossypol and the relationship to its metabolic fate in dogs and monkeys (author's translation)]. *Zhongguo Yao Li Xue. Bao*. 1:39-43.

Seidman, A.D., P.P. Rosen, W. Tong, *et al*. (1999). Phase I/II pharmacologic and cytokinetic study of oral gossypol (G) in patients (pts) with anthracycline (A) and taxane (T) refractory metastatic breast cancer (MBC). *Proc Amer Soc Clin Oncol*. Vol. 18: A426.

Shelley, M.D., L. Hartley, R.G. Fish, *et al*. (1999). Stereo-specific cytotoxic effects of gossypol enantiomers and gossypolone in tumour cell lines. *Cancer Lett.* 135(2):171-180.

Stein, R.C., A.E. Joseph, S.A. Matlin, *et al.* (1992). A preliminary clinical study of gossypol in advanced human cancer. *Cancer Chemother Pharmacol.* 30:480-482.

Tang, X.C., M.K. Zhu, and Q.X. Shi. (1980). [Comparative studies on the absorption, distribution and excretion of 14C-gossypol in four species of animals (author's translation)]. *Yao Xue Xue Bao* 15:212-217.

Wu, D.F., Y.W. Yu, Z.M. Tang, and M.Z. Wang. (1986). Pharmacokinetics of (+/-)-, (+)-, and (-)-gossypol in humans and dogs. *Clin Pharmacol. Ther* 39:613-618.

Xu L., D. Yang, S. Wang, *et al.* (2005). (-)-Gossypol enhances response to radiation therapy and results in tumor regression of human prostate cancer. *Mol Cancer Ther* 4(2):197-205.

15.0 INFORMED CONSENT

15.1 Ethical and Legal Considerations

This study will be conducted in accordance with the Declaration of Helsinki and according to the guidelines in the attached appendices and in compliance with all applicable laws and regulations of the locale where the study is conducted.

It is the responsibility of the investigator that the patient is made aware and consent is given that personal information may be scrutinized during audits by competent authorities and properly authorized persons, but that personal information will be treated as strictly confidential and not be publicly available. The investigator is responsible for the retention of the patient log and patient records.

A Phase 2 Study of R-(-)-gossypol (Ascenta's AT-101) in Recurrent Glioblastoma Multiforme

This is a clinical trial (a type of research study). Clinical trials include only patients who choose to take part. Please take your time to make your decision. Discuss it with your family and friends.

You are being asked to take part in this study because you have a brain tumor (glioma), which has grown or has recurred.

WHY IS THIS STUDY BEING DONE?

The purpose of this study is to test the safety and side effects of AT-101 when it is given to patients with brain cancer. AT-101 is an investigational drug, which means that AT-101 is not approved by the United States Food and Drug Administration (FDA) for general use. The FDA does permit its use in studies like this one to determine whether it is safe and effective.

AT-101 is a form of a drug called gossypol. Gossypol has been studied extensively in more than 9,000 people, mostly in China, as a male contraceptive (form of birth control) and for treating endometriosis (a condition in the tissues of the uterus or "womb") and myoma (tumors of the uterus). Gossypol has also been studied as a treatment for cancer. Four (4) studies in the United States and Britain were done, which included about 100 patients. Those studies identified safe doses of gossypol that could be given to cancer patients. Some patients with brain cancer or cancer of the adrenal gland had their tumors shrink in size when they received treatment with gossypol. Although AT-101 has not been studied extensively like gossypol, laboratory studies suggest that the form of gossypol in AT-101 may be a more effective form in treating cancer.

AT-101 is in its early stage of testing on humans. Currently, AT-101 is being studied in research studies of patients with early and advanced cancers. The purpose of these studies is to find the highest dose of AT-101 that can be safely given to patients without causing severe side effects, and to see the effects AT-101 has to shrink or stop these cancers from growing.

This research is being done because currently, there are few effective treatments for this type of cancer. This drug may be active in other types of cancer and we are studying the side effects (good and bad) of AT-101 when given to patients with brain tumors.

HOW MANY PEOPLE WILL TAKE PART IN THIS STUDY?

About 56 people will take part in the study.

WHAT IS INVOLVED IN THE STUDY?

If you take part in this study you will have the following tests and procedures:

Before treatment starts, you will have a complete physical exam, and blood tests. You will have a physical examination including measurement of your height, weight, blood pressure, pulse rate, breathing rate, performance status, which measures the degree of your ability to complete daily activities (e.g., dressing and eating). You will have a complete neurological exam to test your thinking and motor skills. You will also take a brief test called a mini mental status exam, which is a series of questions that evaluate your mental abilities. Additionally, you will have an electrocardiogram (ECG) (a test that produces a graphic picture of the electric activity of your heart). To measure the extent of your brain cancer, you will a have an MRI scan, which is method of obtaining pictures of internal soft bodily tissue through the use of powerful magnets and radio waves.

About 3 tablespoons of blood will be drawn from one of your veins to conduct laboratory tests (e.g., standard blood count including a count of your platelets, and blood chemistries). You will also be asked to give a sample of urine in order to have a pregnancy test if you are a woman that is able to have children.

AT-101 is given in the form of a pill that you take by mouth. You will take AT-101 every day for 21 out of 28 days (3 out of 4 weeks). Twenty-eight days is considered a treatment cycle. You should swallow these pills at least 1 hour (or more) before or at least 1 hour (or more) after a meal. While taking AT-101 you should remember that over-the-counter medications containing divalent cations (i.e. diary products, calcium supplements, or antacids) must be taken 2 hours before or 4 hours after your dose of AT-101. It is important for you to take AT-101 at about the same time every day. You will be given a drug diary and you will be required to record the time and the number of pills you take. Your doctor will instruct you if there are any changes to this routine. If you have side effects that are too severe, your dose of AT-101 may be reduced for safety, or discontinued entirely if necessary.

Your study doctor will give you AT-101 at the clinic. You will be given instructions on how to store your AT-101 pills and when to take them. You will return any empty bottles and/or remaining AT-101 at each visit to your study doctor.

After the second treatment cycle an MRI will be obtained. If this MRI shows that the tumor size is the same or smaller, you will begin another 28-day (four-week) cycle of treatment. An MRI will be obtained before every other treatment cycle (every 8 weeks) to see if your tumor is growing. During the study, physical exams, neurologic exams and ECGs will be repeated. Routine blood tests will be done. If at any point your blood counts are low, these blood tests will be repeated more often.

Two extra blood samples will be drawn for research purposes as a mandatory part of this study. One blood sample will be drawn immediately before your first dose of AT-101 and one sample will be drawn 1 week after starting therapy (Day 8 of Cycle 1). These blood samples will be used to measure protein levels in your blood. These blood samples are for research purposes only and would not be drawn as a part of routine care.

As a part of this study we will collect samples of your tumor tissue for research purposes. Your doctor will check to see if some of your tumor tissue is available. If tumor tissue is available, some of the tumor tissue that was taken during your surgery or biopsy will be sent to a lab for analysis. The tissue samples will be used to characterize the immunochemistry and molecular biology of your tumor. Glioblastoma multiforme can be very

different in each patient. We hope to learn about the characteristics of your tumor. We will use the information we collect about this kind of tumor to help us better treat patients with glioblastoma multiforme in the future. If your tumor tissue is available it will be required as part of this study.

HOW LONG WILL I BE IN THE STUDY?

Treatment with AT-101 will be continued on the same schedule unless your tumor grows, or you have side effects that warrant discontinuation, or you desire to stop treatment. The researcher may decide to take you off this study if your doctor thinks it will be in your best interest, your condition worsens, or new information becomes available.

Your participation in your clinical trial is completely voluntary. You can stop participating at any time. If you decide not to participate, or wish to withdraw your consent to participate in this treatment at any time, it will in no way affect your regular treatments or medical care. If you stop participating in this study you may be asked to return to the clinic for a final evaluation. However, if you decide to stop participating in the study, we encourage you to talk to the researcher and visit your regular doctor first.

WHAT ARE THE RISKS OF THE STUDY?

While on the study, you are at risk for these side effects. You should discuss these with the researcher and/or your regular doctor. There also may be other side effects that we cannot predict. Other drugs will be given to make the side effects less serious and uncomfortable.

Many side effects go away shortly after the AT-101 is stopped, but in some cases side effects can be serious or long-lasting or permanent or fatal.

Likely Side Effects (> 20% of patients experience these)**:**
- Belly pain
- Diarrhea
- Nausea or the urge to vomit
- Vomiting
- Fatigue or tiredness
- Loss of appetite

Less Likely Side Effects Include (≤ 20% of patients experience these)**:**
- Lack of enough red blood cells (anemia)
- Swelling or feeling of fullness and tightness in the abdomen (belly)
- Constipation
- Dry mouth
- Heartburn
- Inflammation (swelling and redness) of the stomach lining
- Gas in the intestinal (bowel) wall
- Partial or complete blockage of the small and/or large bowel. Ileus is a functional rather than actual blockage of the bowel.
- Blockage of the small bowel
- Swelling of the extremities (arms and/or legs)
- Fever

- Infection
- Increased blood level of a liver enzyme (ALT/SGPT)
- Increased blood level of a liver or bone enzyme (alkaline phosphatase)
- Increased blood level of a liver enzyme (AST/SGOT)
- Increased blood level of a liver pigment (bilirubin) often a sign of liver problems
- Increased blood level of a heart muscle protein (troponin I) indicating damage to the heart muscle
- Increased blood level of a heart muscle protein (troponin T) indicating damage to the heart muscle
- Increased blood level of creatinine (a substance normally eliminated by the kidneys into the urine)
- Weight loss
- Decrease in the total number of white blood cells (leukocytes)
- Dehydration (when your body does not have as much water and fluid as it should)
- Decreased blood level of calcium
- Decreased blood level of potassium
- Decreased blood level of magnesium
- Back pain
- Leg and/or arm pain
- Dizziness (or sensation of lightheadedness, unsteadiness, giddiness, spinning or rocking)
- Taste changes
- Headache or head pain
- Difficulty sleeping or falling asleep
- Decreased sexual desire
- Complete absence of sperm in semen
- Irregular menstrual periods
- Cough
- Shortness of breath
- Dry skin
- Itching
- Skin rash with the presence of macules (flat discolored area) and papules (raised bump)

Side effects due to blood drawing may include: pain, bruising, bleeding or other discomfort at the blood drawing site. Anemia (low red blood cells), fainting or infection at the blood drawing site may occur (very unlikely).

Reproductive risks: Because there may be a risk of birth defects if a fetus is exposed to AT-101, you should not use this drug if you and your partner are planning to have a baby during this study or two months following the end of the study. A barrier method of birth control (i.e. condom) must be used during the study and for eight (8) weeks after your last dose of study drug. You should not nurse a baby while on this study. Ask about counseling and more information about preventing pregnancy.

Financial risks: There may be extra costs with this treatment. Some of the costs may not be covered by the hospital or the insurance company. We encourage you to work closely with your insurance company to find out exactly what is covered for this research study.

For more information about risks and side effects, ask the researcher or contact ________________________.

ARE THERE BENEFITS TO TAKING PART IN THE STUDY?

If you agree to take part in this study, there may or may not be direct medical benefit to you. The benefits of AT-101 treatment to patients with brain tumors is unknown. We hope the information learned from this study will benefit other patients with brain tumors in the future.

WHAT OTHER OPTIONS ARE THERE?

Should your disease become worse, or if you have severe side effects, this therapy may be discontinued and other means of treating you will be discussed. You may choose at this time, or any time in the future to have no further therapy; other than care for relief of your symptoms (supportive care). You may also consider other treatments for your disease such as further surgery, radiation therapy or chemotherapy. Other research options may also be available to you.

Please talk to you doctor about these and other options.

WHAT ABOUT CONFIDENTIALITY?

This section tells you what information about you may be used and given out in this study and who may give and receive the information. By signing this consent form, you agree that health information that identifies you may be used and/or given out as needed in this study. _________________ has a policy to protect health information that may identify you. Federal and state laws also protect your privacy.
______________________ has procedures in place to support these policies and laws.

a. What information about you may be used or given out in this study?

Information that identifies you and relates to your health or medical condition may be used or given out in this study. Information that identifies you can include your name, address, telephone number, date of birth, Social Security number and other details about you. Information that relates to your health or medical condition includes:

Information obtained from the activities and procedures outlined in this consent form, which may include:

i) things done to see if you can join this study, such as physical examinations, blood and urine tests and any other information that you share with us, including information about your health history and your family health history; and

ii) information obtained during the study, such as how you respond to the study activities or procedures, information we learn in study visits, phone calls, surveys, physical examinations, blood and urine tests, x-rays and other tests and any other medical information we learn from you as a participant in this study.

b. Who may use and give out information about you?

Some people may see your health information and may give out your health information during this study. These include the researcher and the research staff, the institutional review boards and their staff, legal counsel, audit and compliance staff, officers of the organization and other people who need to see the information to help this study or make sure it is being done as it should.

c. Who may see your health information?
Other organizations may see your health information during this study. These include:

- Governmental entities that have the right to see or review your health information, such as the Office of Human Research Protections and the Food and Drug Administration
- Doctors and staff at other places that are participating in this study
- The pharmaceutical collaborators, Ascenta Therapeutics, Inc.
- The sponsor of this study and people that the sponsor my contract with for this study. The name of the sponsor is CTEP/NCI (National Cancer Institute).
- The Contract Research Organization.
- The Data Safety Monitoring Board
- An outside institutional review board
- ABTC CNS Consortium

d. Why will this information be used and given out?
Your information will be used and given out to carry out this study and to evaluate the results of this study.

e. What if you decide not to give your permission to use and give out your health information?
You do not have to give your permission to use or give out your health information. However, if you do not give permission, you may not participate in this study.

f. May you withdraw or cancel your permission?
You may cancel your agreement to allow your health information to be used or given out at any time by sending a written notice to the Institutional Review Board Office, ____________________________________. If you do this, you are leaving this study. If you leave this study, no new health information about you will be gathered after that date. However, information gathered before that date may be used or given out if it is needed for this study or any follow-up for this study.

g. Is your health information protected after it has been given to others?
If your health information is given to someone not covered by these policies and laws, that information may no longer be protected, and may be used or given out without your permission.

h. Does this consent form have an end date?
This authorization to use and give out health information continues until the end of this study and any necessary data analysis follow-up activities for this study.

A) What is the role of the IRB?
Research studies involving human volunteers are reviewed by an Institutional Review Board (IRB). The IRB is made up of doctors, nurses, scientists, non-scientists and people from the community. The IRB is responsible for protecting participant's rights.

WHAT ARE THE COSTS?

Taking part in this study may lead to added costs to you or your insurance company. Some of the costs of the blood tests and brain scans may not be covered by your insurance company. We encourage you to work closely with your insurance company to find out exactly what is covered for this research study.

In the case of injury or illness resulting from this study, emergency medical treatment is available but will be provided at the usual charge. No funds have been set aside to compensate you in the event of injury.

You or your insurance company will be charged for continuing medical care and/or hospitalization.

The study agent, AT-101, will be provided free of charge by the Division of Cancer Treatment and Diagnosis, NCI, while you are participating in this study. However, if you should need to take the study agent much longer than is usual, it is possible that the supply of free study agent that has been supplied to the NCI could run out. If this happens, your study doctor will discuss with you how to obtain additional drug from the manufacturer and you may be asked to pay for it.

You will receive no payment for taking part in this study.

WHAT ARE MY RIGHTS AS A PARTICIPANT?

Taking part in this study is voluntary. You may choose not to take part or may leave the study at any time. Leaving the study will not result in any penalty or loss of benefits to which you are entitled.
We will talk to you about new information that may affect your health, welfare, or willingness to stay in this study.

WHOM DO I CALL IF I HAVE QUESTIONS OR PROBLEMS?

For questions about the study or a research-related injury, contact the researcher __________________ (NAME{S}) at __________________ (TELEPHONE NUMBER) .

For questions about your rights as a research participant, contact the__________________ (NAME OF CENTER) Institutional Review Board (which is a group of people who review the research to protect your rights) at __________________ (TELEPHONE NUMBER).

WHERE CAN I GET MORE INFORMATION?

You may call the NCI's Cancer Information Service at

1 800 4 CANCER (1 800 422 6237) or TTY: 1 800 332 8615

Visit the NCI's Web sites

cancerTrials: comprehensive clinical trials information
http://cancertrials.nci.nih.gov.

CancerNet: accurate cancer information including PDQ
http://cancernet.nci.nih.gov.

You will get a copy of this form. You may also request a copy of the protocol (full study plan).

SIGNATURE

I agree to take part in this study.

Participant ________________________________

Date _____________________

Investigator ________________________________

Date _____________________

Witness ___________________________________

Date ____________________

National Institutes of Health
National Cancer Institute

Division of Cancer Treatment and Diagnosis
Cancer Therapy Evaluation Program

Please Print or Type

Protocol No.: ____________ **Patient ID No.:** ____________________ **Course No.:** __________

Date of Event		Category/Toxicity	Grade	*Attribution
	Yes / No	**ALLERGY/IMMUNOLOGY**		
______		Allergic reaction/hypersensitivity (including drug fever)	1 2 3 4 5	1 2 3 4 5
______		______	1 2 3 4 5	1 2 3 4 5
______		______	1 2 3 4 5	1 2 3 4 5
	Yes / No	**AUDITORY/HEARING**		
______		______	1 2 3 4 5	1 2 3 4 5
	Yes / No	**BLOOD/BONE MARROW**		
______		Hemoglobin (Hgb)	1 2 3 4 5	1 2 3 4 5
______		Leukocytes (total WBC)	1 2 3 4 5	1 2 3 4 5
______		Neutrophils/granulocytes (ANC/AGC)	1 2 3 4 5	1 2 3 4 5
______		Platelets	1 2 3 4 5	1 2 3 4 5
______		Transfusion: platelets	3 4 5	1 2 3 4 5
______		Transfusion: pRBCs	3 4 5	1 2 3 4 5
______		______	1 2 3 4 5	1 2 3 4 5
______		______	1 2 3 4 5	1 2 3 4 5
	Yes / No	**CARDIOVASCULAR (ARRHYTHMIA)**		
______		______	1 2 3 4 5	1 2 3 4 5
______		______	1 2 3 4 5	1 2 3 4 5
	Yes / No	**CARDIOVASCULAR (GENERAL)**		
______		Cardiac-ischemia/infarction	1 2 3 4 5	1 2 3 4 5
______		Cardiac left ventricular function	1 2 3 4 5	1 2 3 4 5
______		Hypotension	1 2 3 4 5	1 2 3 4 5
______		______	1 2 3 4 5	1 2 3 4 5
______		______	1 2 3 4 5	1 2 3 4 5
	Yes / No	**COAGULATION**		
______		______	1 2 3 4 5	1 2 3 4 5
______		______	1 2 3 4 5	1 2 3 4 5
	Yes / No	**CONSTITUTIONAL SYMPTOMS**		
______		Fatigue	1 2 3 4 5	1 2 3 4 5
______		Weight loss	1 2 3	1 2 3 4 5
______		______	1 2 3 4 5	1 2 3 4 5
______		______	1 2 3 4 5	1 2 3 4 5

*Attribution Key:

Unrelated	1	The adverse event (toxicity) is not related to the investigational agent(s).
Unlikely	2	The adverse event (toxicity) is doubtfully related to the investigational agent(s).
Possible	3	The adverse event (toxicity) may be related to the investigational agent(s).
Probable	4	The adverse event (toxicity) is likely related to the investigational agent(s).
Definite	5	The adverse event (toxicity) is clearly related to the investigational agent(s).

Date of Event		Category/Toxicity	Grade	*Attribution
	Yes / No	**DERMATOLOGY/SKIN**		
______		Alopecia	1 2	1 2 3 4 5
______		Rash/desquamation	1 2 3 4 5	1 2 3 4 5
______		Urticaria (hives, welts, wheals)	1 2 3	1 2 3 4 5
______		______	1 2 3 4 5	1 2 3 4 5
______		______	1 2 3 4 5	1 2 3 4 5
	Yes / No	**ENDOCRINE**		
______		______	1 2 3 4 5	1 2 3 4 5
	Yes / No	**GASTROINTESTINAL**		
______		Anorexia	1 2 3 4 5	1 2 3 4 5
______		Dehydration	1 2 3 4 5	1 2 3 4 5
______		Diarrhea	1 2 3 4 5	1 2 3 4 5
______		Dysphagia, esophagitis, odynophagia (painful swallowing)	1 2 3 4 5	1 2 3 4 5
______		Nausea	1 2 3	1 2 3 4 5
______		Stomatitis/pharyngitis (oral/pharyngeal mucositis)	1 2 3 4 5	1 2 3 4 5
______		Vomiting	1 2 3 4 5	1 2 3 4 5
______		______	1 2 3 4 5	1 2 3 4 5
______		______	1 2 3 4 5	1 2 3 4 5
	Yes / No	**HEMORRHAGE**		
______		Hemorrhage/bleeding with grade 3 or 4 thrombocytopenia	1 3 4 5	1 2 3 4 5
______		Hemorrhage/bleeding without grade 3 or 4 thrombocytopenia	1 3 4 5	1 2 3 4 5
______		______	1 2 3 4 5	1 2 3 4 5
______		______	1 2 3 4 5	1 2 3 4 5
	Yes / No	**HEPATIC**		
______		Bilirubin	1 2 3 4 5	1 2 3 4 5
______		GGT	1 2 3 4 5	1 2 3 4 5
______		SGOT (AST)	1 2 3 4 5	1 2 3 4 5
______		SGPT (ALT)	1 2 3 4 5	1 2 3 4 5
______		______	1 2 3 4 5	1 2 3 4 5
______		______	1 2 3 4 5	1 2 3 4 5
	Yes / No	**INFECTION/FEBRILE NEUTROPENIA**		
______		Catheter-related infection	1 2 3 4 5	1 2 3 4 5
______		Febrile neutropenia (fever of unknown origin without clinically or microbiologically documented infection. (ANC <1.0 x 10^9/L, fever > 38.5)	3 4 5	1 2 3 4 5
______		Infection (documented clinically or microbiologically) with grade 3 or 4 neutropenia. (ANC < 1.0 x 10^9/L)	3 4 5	1 2 3 4 5
______		Infection without neutropenia	1 2 3 4 5	1 2 3 4 5
______		______	1 2 3 4 5	1 2 3 4 5
______		______	1 2 3 4 5	1 2 3 4 5

*Attribution Key:			
	Unrelated	1	The adverse event (toxicity) is not related to the investigational agent(s).
	Unlikely	2	The adverse event (toxicity) is doubtfully related to the investigational agent(s).
	Possible	3	The adverse event (toxicity) may be related to the investigational agent(s).
	Probable	4	The adverse event (toxicity) is likely related to the investigational agent(s).
	Definite	5	The adverse event (toxicity) is clearly related to the investigational agent(s).

Date of Event		Category/Toxicity	Grade	*Attribution
	Yes / No	**LYMPHATICS**		
______		______	1 2 3 4 5	1 2 3 4 5
	Yes / No	**METABOLIC/LABORATORY**		
______		______	1 2 3 4 5	1 2 3 4 5
______		______	1 2 3 4 5	1 2 3 4 5
	Yes / No	**MUSCULOSKELETAL**		
______		______	1 2 3 4 5	1 2 3 4 5
______		______	1 2 3 4 5	1 2 3 4 5
	Yes / No	**NEUROLOGY**		
______		Neuropathy-cranial	2 3 4 5	1 2 3 4 5
______		Neuropathy-motor	1 2 3 4 5	1 2 3 4 5
______		Neuropathy-sensory	1 2 3 4 5	1 2 3 4 5
______		______	1 2 3 4 5	1 2 3 4 5
______		______	1 2 3 4 5	1 2 3 4 5
	Yes / No	**OCULAR/VISUAL**		
______		______	1 2 3 4 5	1 2 3 4 5
	Yes / No	**PAIN**		
______		______	1 2 3 4 5	1 2 3 4 5
	Yes / No	**PULMONARY**		
______		______	1 2 3 4 5	1 2 3 4 5
	Yes / No	**RENAL/GENITOURINARY**		
______		Creatinine	1 2 3 4 5	1 2 3 4 5
______		______	1 2 3 4 5	1 2 3 4 5
______		______	1 2 3 4 5	1 2 3 4 5
	Yes / No	**SECONDARY MALIGNANCY**		
______		______	1 2 3 4 5	1 2 3 4 5
	Yes / No	**SEXUAL/REPRODUCTIVE FUNCTION**		
______		______	1 2 3 4 5	1 2 3 4 5

*Attribution Key:

Unrelated	1	The adverse event (toxicity) is not related to the investigational agent(s).
Unlikely	2	The adverse event (toxicity) is doubtfully related to the investigational agent(s).
Possible	3	The adverse event (toxicity) may be related to the investigational agent(s).
Probable	4	The adverse event (toxicity) is likely related to the investigational agent(s).
Definite	5	The adverse event (toxicity) is clearly related to the investigational agent(s).
